# NADPH-Oxidase Derived Hydrogen Peroxide and Irs2b Facilitate Re-oxygenation-Induced Catch-Up Growth in Zebrafish Embryo

**DOI:** 10.3389/fendo.2022.929668

**Published:** 2022-07-01

**Authors:** Ayaka Zasu, Futa Hishima, Marion Thauvin, Yosuke Yoneyama, Yoichiro Kitani, Fumihiko Hakuno, Michel Volovitch, Shin-Ichiro Takahashi, Sophie Vriz, Christine Rampon, Hiroyasu Kamei

**Affiliations:** ^1^ Faculty of Biological Science and Technology, Institute of Science and Engineering, Kanazawa University, Noto, Japan; ^2^ Center for Interdisciplinary Research in Biology (CIRB), Collège de France, Centre national de la recherche scientifique (CNRS), Institut National de la Santé et de la Recherche Médicale (INSERM), Paris Sciences et Lettres (PSL) Research University, Paris, France; ^3^ Sorbonne Université, Ecole Doctorale 515-Complexité du Vivant, Paris, France; ^4^ Departments of Animal Sciences and Applied Biological Chemistry, Graduate School of Agriculture and Life Sciences, The University of Tokyo, Tokyo, Japan; ^5^ Institute of Research, Tokyo Medical and Dental University, Tokyo, Japan; ^6^ Noto Marine Laboratory, Division of Marine Environmental Studies, Institute of Nature and Environmental Technology, Kanazawa University, Noto, Japan; ^7^ Department of Biology, École Normale Supérieure, Paris Sciences et Lettres (PSL) Research University, Paris, France; ^8^ Laboratoire des BioMolécules (LBM), Département de Chimie, Sorbonne Université, École Normale Supérieure, Paris Sciences et Lettres (PSL) University, Sorbonne Université, Centre national de la recherche scientifique (CNRS), Paris, France; ^9^ Université Paris-Cité, Faculty of Sciences, Paris, France

**Keywords:** zebrafish, hypoxia, re-oxygenation, hydrogen peroxide, NADPH-oxidase, catch-up growth, insulin-like growth factor, insulin receptor substrate 2

## Abstract

Oxygen deprivation induces multiple changes at the cellular and organismal levels, and its re-supply also brings another special physiological status. We have investigated the effects of hypoxia/re-oxygenation on embryonic growth using the zebrafish model: hypoxia slows embryonic growth, but re-oxygenation induces growth spurt or *catch-up* *growth*. The mitogen-activated kinase (MAPK)-pathway downstream insulin-like growth factor (IGF/Igf) has been revealed to positively regulate the re-oxygenation-induced catch-up growth, and the role of reactive oxygen species generated by environmental oxygen fluctuation is potentially involved in the phenomenon. Here, we report the role of NADPH-oxidase (Nox)-dependent hydrogen peroxide (H_2_O_2_) production in the MAPK-activation and catch-up growth. The inhibition of Nox significantly blunted catch-up growth and MAPK-activity. Amongst two zebrafish insulin receptor substrate 2 genes (*irs2a* and *irs2b*), the loss of *irs2b*, but not its paralog *irs2a*, resulted in blunted MAPK-activation and catch-up growth. Furthermore, *irs2b* forcedly expressed in mammalian cells allowed IGF-MAPK augmentation in the presence of H_2_O_2_, and the *irs2b* deficiency completely abolished the somatotropic action of Nox in re-oxygenation condition. These results indicate that redox signaling alters IGF/Igf signaling to facilitate hypoxia/re-oxygenation-induced embryonic growth compensation.

## Introduction

When growing animals encounter adverse conditions, they are prone to reduce growth rate (1). However, intriguingly, upon removing the harsh condition, the stunted animals restart growth with accelerated progression to rapidly reach the original growth level, termed “*catch-up growth*” (2, 3). In humans, this compensation phenomenon often occurs in newborns who experienced intrauterine growth restriction (IUGR) (3–5). Since the catch-up growth is known to associate with adult-onset diseases (such as type 2 diabetes, obesity, and cardiovascular diseases) affecting multiple organs in the later life of IUGR infants (6), keen attention has been paid to the underlying molecular mechanisms in the etiology. On the other hand, though the rapid growth process should also have an intimate connection with the diathesis, we still know little about the molecular basis of the growth spurt. In mammalian models, inhibition of cellular senescence in the epiphyseal cartilage of long bone has been suggested as a key to catch-up growth (7). Nevertheless, the catch-up growth also occurs even in non-mammalian early embryos (2), which do not show a senescence process, and in which the chondrogenic differentiation is still in progress. Thus, it is assumed that this phenomenon has an unknown systemic mechanism(s).

An experimental model of hypoxia-induced growth retardation and the following re-oxygenation-induced catch-up growth has been developed in zebrafish embryos (2). Hypoxia reduced growth-promoting signals such as insulin-like growth factor (IGF/Igf) signaling, but re-oxygenation restored it (2). The insulin and IGF/Igf-signaling activate two major downstream signaling pathways, such as PI3K- and MAPK-pathways (8, 9). Notably, the Igf-MAPK-pathway is responsible for growth acceleration in the zebrafish model of catch-up growth (2). The insulin receptor substrate (IRS/Irs) is an intracellular Igf-signaling mediator that transduces type-I IGF/Igf-receptor (IGF1R/Igf1r) activation to both PI3K- and MAPK-pathways (8, 9). Four IRS/Irs genes (*IRS/Irs1-4*) are known from fish to primates, and most animal genomes retain *IRS1/Irs1* and *IRS2/Irs2* genes (10). The physiological significance of IRS/Irs proteins has been studied primarily using gene knockout mice. Specific to growth, defects in the *Irs1* gene cause marked growth retardation from the embryonic stage (11). Meanwhile, mice lacking the *Irs2* gene do not show a significant growth-inhibitory phenotype. Instead, *Irs2* deficient mice show severe hepatic insulin resistance, impaired beta-cell proliferation, insulin secretion, and diabetic symptoms caused outside of growth (12, 13). However, a recent knockout study has shown that deletion of the *Irs2* gene in rats causes significant growth retardation (14). In addition, the role of Irs1 was examined in a catch-up growth model using zebrafish and found it was responsible for the survival of multipotent cells vital for the growth spurt (15). On the other hand, we have not examined Irs affecting the activation of the Mapk-pathway during catch-up growth or the role of Irs2, leaving room for related studies.

Oxygen is an indispensable molecule for the anabolism and efficient generation of cellular energy or ATP in most cells (16, 17). In addition to its anabolic roles, oxygen turns into reactive molecular species such as superoxide- and peroxy-radicals, and their derivatives (18, 19). Thus, oxygen and these reactive molecular species lay in the inevitable nexus. Indeed, an extreme decrease in oxygen concentration followed by its rapid restoration causes a significant change in the cells’ generation of oxidative compounds or reactive oxygen species (ROS) (20). Since *in vivo* local oxygen consumption and its level alters according to motor activity and local metabolism, animals are constantly exposed to dynamic changes in oxygen availability and ROS generation. Hydrogen peroxide (H_2_O_2_) is a common derivative of superoxide radicals and peroxide ions; its fundamental importance in numerous biological events is widely documented (21, 22). The H_2_O_2_ triggers cellular injury, but it also acts as an essential cellular signal to induce tissue regeneration (23, 24). It has also been reported that H_2_O_2_ increases the expression and tyrosine phosphorylation of Irs2 (25, 26), suggesting that H_2_O_2_ may contribute to the re-oxygenation-induced Igf-signaling *via* Irs2. One of the major family of enzymes involved in H_2_O_2_-generation is NADPH-oxidases or Nox (19, 21, 22). Nox enzymes are transmembrane protein that metabolizes oxygen and water to produce H_2_O_2_, either directly (Nox4, Duox1 and 2) or in combination with superoxide dismutase (SOD) (Nox1, 2, 3 and 5). After production in the extracellular compartment, H_2_O_2_ is rapidly imported into cells by dedicated aquaporins. Therefore, the amount of H_2_O_2_ in the vicinity of the cell membrane, where the Igf1r-Irs signaling ignites, would be influenced by environmental oxygen level and Nox activity. Previous studies also indicated that Nox molecules generated the membrane-localized H_2_O_2_ influenced IGF-signaling both *in vitro* and *in vivo* (27, 28).

This study investigated the functional nexus between Nox and Irs2 in the re-oxygenation-induced catch-up growth. First, we examined changes in the H_2_O_2_ levels in zebrafish embryos during hypoxia and subsequent re-oxygenation, using specific H_2_O_2_-sensing probes that enable us to live-image H_2_O_2_. Next, the contribution of Nox-derived H_2_O_2_ to catch-up growth was investigated. In addition, we identified an *irs2, irs2b*, which played a vital role in catch-up growth in conjunction with H_2_O_2_ generated by Nox. Our data suggest that the Nox-mediated H_2_O_2_ brings a context-dependent MAPK-activating function to the Irs2b, initiating re-oxygenation-induced catch-up growth. This study would help understand the relationship between redox signaling and growth promotion in developing embryos.

## Materials and Methods

### Chemicals

Chemicals and reagents were purchased from Fujifilm-Wako (Tokyo, Japan) and Nacalai Tesque (Kyoto, Japan) unless noted otherwise. Trizol reagent, reverse transcriptase, and oligonucleotide primers were purchased from Invitrogen Life Technologies (Invitrogen, Carlsbad, CA, USA).

### Experimental Animals

Adult zebrafish (*Danio rerio*) were kept at around 27-29°C on a 14 hr-light:10 hr-dark cycle and fed twice daily. Natural crosses obtained fertilized eggs which were raised at 28.5°C and staged according to a previous report (15, 29). Embryos were anesthetized in tricaine mesylate (ethyl 3-aminobenzoate methane sulfonate; Sigma-Aldrich Japan, Tokyo). All experiments were conducted under guidelines authorized by the committees on the use and care of animals at Kanazawa University and Collège de France.

### Hypoxia and Re-Oxygenation of the Zebrafish Embryo

As previously reported (30), hypoxic water was prepared by bubbling pure nitrogen gas into the embryo rearing solution. Oxygen concentrations were measured using a dissolved oxygen meter (YSI Model ProODO, YSI Nanotech Japan, Kawasaki, Japan). The amount of dissolved oxygen in the hypoxic water was ≈ 0.85 ± 0.2 mg O_2_/L (approximately 10% of oxygen when the oxygen concentration in normoxic water is 100%: ≈ 8.5 mg O_2_/L). In the experiment, all embryos were kept under normoxia for up to 24 hr post-fertilization (hpf). In the hypoxia/re-oxygenation experiment, embryos shifted to hypoxia from 24 hpf to 32 (or 36) hpf were designated Hypo. Embryos placed in constant normoxia were referred to as Norm. Embryos exposed to hypoxia and then returned to normoxic water were denoted as Reoxy.

### HyPer Imaging and Image Processing

**Figure 1:**
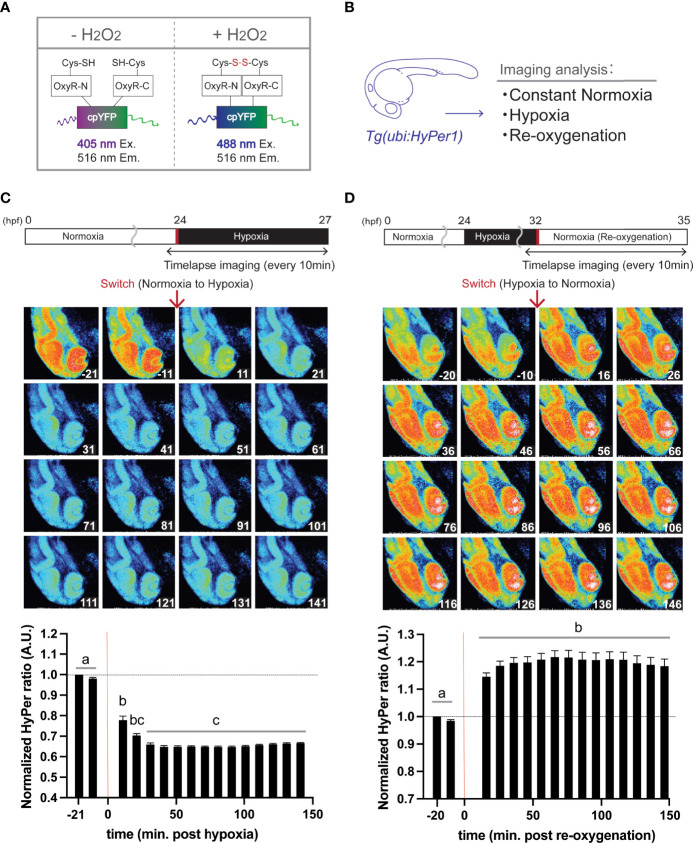
Hypoxia blunts H_2_O_2_ generation, but re-oxygenation regains it in HyPer1 transgenic zebrafish embryo. **(A)** Schematic illustration of fluorometric features of the HyPer1, a specific H_2_O_2_ sensor. **(B)** Experiment summary. The transgenic zebrafish embryos harboring ubiquitous HyPer1 expression were used. The embryos were placed in distinct environments (constant normoxia, hypoxia, and re-oxygenated normoxia) and subjected to imaging analysis. **(C, D)** Changes in H_2_O_2_ levels in zebrafish embryos during the transition from normoxia to hypoxia **(C)** or from hypoxia to normoxia **(D)**. Transgenic fish expressing HyPer1 under the control of a *ubiquitin* gene promoter (*ubi*) were allowed to develop normally for up to 24 hr post-fertilization (hpf), and fluorescence signals were detected approx. 20 and 10 min before each treatment. After that, the rearing water of the embryos was replaced with hypoxic **(C)** or normoxic **(D)** water, and the images were timelapse photographed every 10 min for 2.5 hr after the transitions. The fluorescence intensity was analyzed in each image. The timing of the red arrows indicates the transition. The HyPer1 signal was calculated relative to the signal intensity of the first photo. Data are mean ± SE of 7 independent experiments. Values marked with different letters (a, b, c) are significantly different from each other (*P<0.05*), but values marked with common letters (b and bc; bc and c) are not significantly different from each other (*P>0.05*).

To measure the amount of H_2_O_2_ in the cell, we used HyPer, a protein whose fluorescence property changes with H_2_O_2_. The HyPer protein has OxyR-domains on both the N- and C-terminal sides of engineered YFP protein. These two OxyR-domains are highly sensitive to H_2_O_2_ to form a disulfide bond between two cysteine (Cys) residues in the OxyR domain, resulting in conformational and fluorometric changes (31) (**Figure 1A**). Transgenic embryos harboring the coding sequence of HyPer1 under the *ubiquitin* gene promoter designated as *Tg(ubi:Hyper1)* (18) were anesthetized in tricaine solution and embedded in low-melting agarose (0.8%). Imaging was performed with a CSU-W1 Yokogawa spinning disk coupled to a Zeiss Axio Observer Z1 inverted microscope equipped with a sCMOS Hamamatsu camera and a 25× (Zeiss 0.8 Imm DIC WD: 0.57 mm) oil objective. DPSS 100 mW 405 nm and 150 mW 491 nm lasers and a 525/50 bandpass emission filter were used for HyPer imaging. To calculate the HyPer ratio, images were treated as previously described (32).

### Analysis of H_2_O_2_ Level During Reoxy Condition

The OxiVision™ Green hydrogen peroxide sensor (OVG, CosmoBio, Tokyo, Japan), a fluorescent dye that selectively reacts with H_2_O_2_, was used to detect H_2_O_2_ in the fish embryo and the culture cell. For the use in the fish embryo, the 24 hpf live specimens were exposed to hypoxia with OVG (10 μM) for 8 hr. After that, we measured the fluorescence intensity at 5 to 10 min after returning to the Reoxy condition. Then, the OVG signal was visualized under the fluorescence stereomicroscope system (Leica M165 FC with the DFC6200 camera, Leica microsystems, Tokyo, Japan) through an eGFP filter. For the use in the cell culture experiment, 5 μM of OVG was added after two washes of human embryonic kidney (HEK) 293T cells (approximately 70-90% confluent) seeded in a p35 dish with Hoechst 33342 (Invitrogen; 1:20,000) in serum-starved medium containing 0.1% BSA. The cells were incubated in a 5% CO_2_ incubator under normoxia or hypoxia. According to the previous report (33), hypoxia for the cells was carried out using a hypoxic chamber containing nitrogen gas. For the re-oxygenation experiment, the cells were incubated in a chamber for 12-14 hr; then, immediately after being taken out of the hypoxic chamber, the timelapse imaging was performed with an inverted fluorescence microscope (BZ9000, Keyence, Tokyo, Japan). The Fiji software calculated the strength of the OVG signals in the fluorescent images.

### Inhibition of Nox and Co-Treatment With H_2_O_2_

As a specific inhibitor of Nox, we used VAS2870 (Enzo Life Sciences, NY, USA), whose efficiency in zebrafish embryos was previously reported (18). The effect of VAS2870 on embryo growth was also analyzed by adding H_2_O_2_ (100-1000 nM) to the rearing water. The concentration of H_2_O_2_ in the rearing water remained relatively stable up to 8 hr after addition but was primarily lost by 24 hr after addition, so the rearing water containing newly adjusted H_2_O_2_ was used every 8 hr as in the case of VAS2870.

### Growth Rate Measurement and Relative Growth Rate Calculation

Embryonic body growth was evaluated by HTA (Head-Trunk Angle) as previously described (15, 29). Growth rate (dh/dt) was obtained by the following equation: dh/dt = (hn-h0) / (tn-t0); h0: HTA at initial time point (t0), hn: HTA at end time point (tn). The relative growth rate was expressed as the percentage of the control group.

### Immunoblot Analysis

Immunoblot (IB) analysis was performed as previously reported (2, 15). Antibodies used for IB were purchased from Cell Signaling Technology (CST-Japan, Tokyo, Japan). Anti-phospho-Akt antibody (9271: phosphorylated at Ser473) was diluted 1:500, and total-Akt (9272), anti-phospho-Erk1/2 (9101: phosphorylated at Thr202, Tyr204) and total-Erk1/2 (137F5) were all diluted 1:1,000. Protein concentrations were quantified with a BCA protein assay kit (Pierce Biotechnology, Rockford, IL, USA) and subjected to SDS-PAGE. After the proteins were separated by SDS-PAGE, they were blotted on to PVDF membrane (Millipore, Darmstadt, Germany). The membrane was treated with each antibody for IB analysis.

### Molecular and Functional Evaluation of Zebrafish Irs2 Proteins

The cDNAs encoding full-length CDSs of zebrafish Irs2a and Irs2b were cloned using the primers (No. 1-4, **Table S1**) and high-fidelity DNA polymerase (Phusion polymerase, Thermo Fisher Scientific-JP, Tokyo, Japan). The zebrafish DNA sequence database (GRCz9) in Ensembl (http://asia.ensembl.org/Danio_rerio/Info/Index) was used for designing cloning primers. The phylogenetic analysis was done using the Geneious Prime software. The amplified cDNAs were subcloned into the pCS2-FLAG vector after the sequential digestion with *EcoR*I and *Xho*I as previously described (15). Recombinant FLAG-tagged zebrafish Irs2 and human IRS2 proteins were expressed in HEK293T cells. Cells were cultured in high-glucose Dulbecco’s modified Eagle’s medium (Nissui, Tokyo, Japan) with 10% fetal bovine serum (Sigma, St. Louis, MO, USA), 2 mM glutamine, and nonessential amino acids. Polyethyleneimine (Polyscience Inc; Warrington, PA, USA) was used to introduce each expression plasmid into the cells. One day after transfection, cells were incubated with a serum-free medium for 12 hr. One hour before IGF1 stimulation, BMS754807 (Selleck Chemicals, Boston, MA 0.5 μM) were applied to block insulin receptor (IR)/IGF1R. Cells were then stimulated with 100 ng/mL IGF1 for 5 min, and a lysis buffer containing protease inhibitor cocktail and phosphatase inhibitor mix was used to treat the cells and collect the lysate. Equal amounts of protein were used for immunoprecipitation (IP) and IB analyses. IP was performed as described previously (15). The IGF1 was a kind gift from Dr. Toshiaki Ohkuma (Fujisawa Pharmaceutical Co., Osaka, current Astellas Pharma Inc., Tokyo, Japan).

### Quantitative Real-Time RT-PCR

Total RNA was extracted using Trizol reagent, and the 1^st^ strand cDNA was synthesized using Super script II reverse transcriptase, according to the manufactures instruction. qRT-PCR was performed using TB Green Premix Ex Taq (Tli RNase H Plus, Takara Bio, Shiga, Japan) and Applied Biosystems^®^ StepOnePlus™ real-time PCR system (Thermo Fisher Scientific, Waltham, MA, USA) as described previously (15). The primers used for the assay (No. 5-10; 17-40) are listed in **Table S1**. The specificity of PCR was confirmed by denaturation curve analysis, and PCR products were analyzed by electrophoresis to determine the single amplicon.

### Microinjection

Translation block antisense MO against zebrafish *irs2a *mRNA (*irs2a* MO: 5’- CCTTTAAGAGGCGGACTTGCCATAC-3’), against zebrafish *irs2b *mRNA* *(*irs2b* MO: 5’- CGGCGGACTCGCCATTCTCATATGC-3’), and standard control MO (control MO: 5’- CCTCTCTTACCTCAGTTACAATTTATA-3’) were purchased from Gene Tools, LLC (Philomath, OR, USA). The cDNAs encoding the MO target sequence of either zebrafish *irs2a *or* irs2b* was cloned using specific primers (No. 11-14, **Table S1**) and Phusion polymerase, and the amplicons were inserted into the linearized pCS2+Venus vector by In-Fusion^®^ HD cloning kit w/Cloning Enhancer (Takara Bio). Linearized pCS+Venus vector was generated by PCR using specific primers (No. 15-16, **Table S1**) and Phusion polymerase. The MO-target Venus mRNAs (*irs2a* MO target-Venus; *irs2b* MO target-Venus) were made *in vitro* capped RNA transcription (mMessage mMachine: Ambion, TX, USA). MOs were injected into embryos at the same dose (4 ng/embryo). The effects of *irs2a* and *irs2b* translation-inhibition MOs were examined by co-injection of MOs with the capped RNA encoding modified fluorescent protein Venus, which has a MO target sequence. Capped RNAs encoding modified Venus, N-terminal FLAG-tagged zebrafish Irs2b which has incomplete MO target sequence, constitutively active Akt (mouse Akt1 with N-terminal myristoylated signal: myrAkt), and constitutively active Ras (HA-RasV12) were synthesized by mMessage mMachine as previously described (15). Capped RNAs for Venus (250 pg/embryo), FLAG-zebrafish Irs2b (1000 pg/embryo), myrAkt (20 pg/embryo), and HA-RasV12 (5 pg/embryo) were injected into 1-2 cell stage embryos and kept at 28.5°C until sampling.

### Signal Activation Experiments Using H_2_O_2_ and IGF1 in HEK293T Cells

HEK293T cells overexpressing pCS2-FLAG and pCS2-FLAG-Irs2b were cultured in a serum-starved medium containing 0.1% BSA overnight. The following day, cells were replaced with a medium supplemented with H_2_O_2_ (0.1 mM) and incubated for 10 min, followed by replacement with a medium containing Long-R3 IGF1 (Sigma; 200 ng/mL) and incubation for 15 min. Immediately afterward, when IGF1-stimulation was completed, cells were snap-frozen at -80°C and used for subsequent analysis.

### Statistics

Values are presented as average ± standard deviation (SD) when the data from a single representative experiment are shown, or mean ± standard error of means (SE) when the data are from multiple repetitive experiments. The Student’s t-test was used for comparing two groups. The multiple groups were compared by one-way analysis of variance (ANOVA) followed by Fisher’s least significant difference test. Statistical tests were performed by GraphPad Prism 9.0 (GraphPad, San Diego, CA), and the significance was admitted at *P<0.05*.

## Results

### Changes in H_2_O_2_ Levels in Zebrafish Embryos in Response to Environmental Oxygen

Using transgenic zebrafish genetically engineered to express HyPer1 systemically under the regulation of a ubiquitin promoter (**Figure 1B**), timelapse imaging was performed every 10 min for about 2.5 hr after the transition from normoxia to hypoxia. Analysis of fluorescence intensity revealed that the cellular H_2_O_2_ levels significantly decreased immediately after the Hypo condition (10 min after the transition, signal intensity changed to about 78% of control), and the signal reached its lowest value (64.7%) at 41 min after the transition (**Figure 1C**). In the same way, we analyzed the HyPer1 signals in zebrafish embryos after Reoxy by taking timelapse images every 10 min for about 2.5 hr. The results showed that the HyPer1 signal increased significantly soon after the transition to Reoxy. The signal reached the highest level 66 min after the Reoxy (signal increased by 121.7%) and remained at a steady high level for the remainder of the imaging period (**Figure 1D**).

### H_2_O_2_ Levels in Embryos and Culture Cells Under Normoxic and Re-Oxygenation Conditions

**Figure 2:**
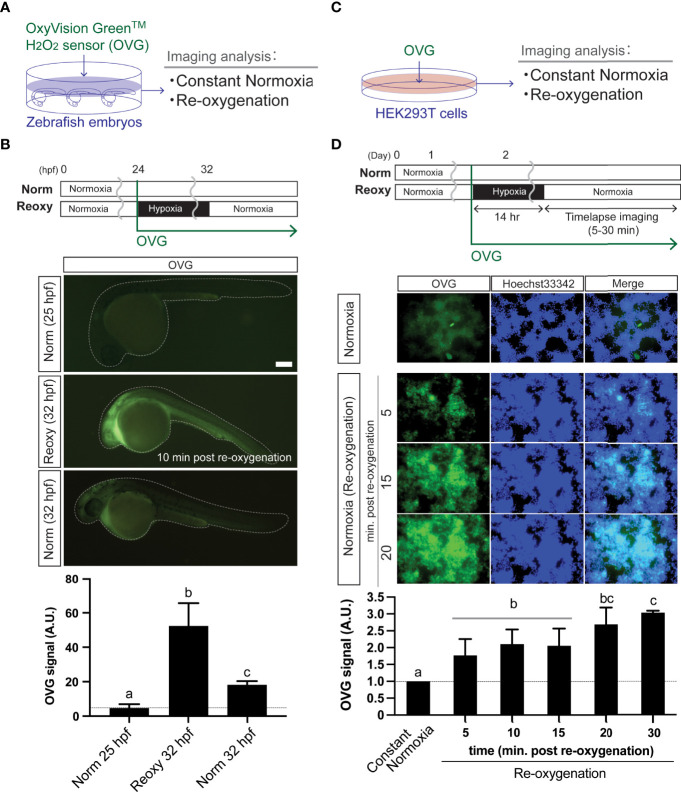
Re-oxygenation increases H_2_O_2_ generation in the zebrafish embryo and mammalian cells. **(A)** Experiment summary. The zebrafish embryos exposed with a specific H_2_O_2_ sensor probe (OxyVision Green™: OVG) were placed in distinct environments (constant normoxia: Norm and re-oxygenation: Reoxy) and subjected to imaging analysis. **(B)** The 24 hpf zebrafish embryos were exposed to hypoxia with 10 μM of OVG for 8 hr. Then, the fluorescence intensity at around 10 minutes after re-oxygenation was measured. The stage-matched (25 hpf Norm) and the chronological age-matched (32 hpf Norm) embryos were also tested for comparison. Bar, 100 μm. The OVG signal was calculated relative to the signal intensity of the 25 hpf Norm embryos. Data are mean ± SE of 5 independent experiments. The different letter denotes statistical significance at *P<0.05*. **(C)** Experiment summary. The human embryonic kidney cells (HEK293T cells) exposed with OVG were placed in Norm or Reoxy condition and subjected to imaging analysis. **(D)** The cells were exposed to 14 hr-long hypoxia with 5 μM of OVG. The OVG signal was calculated relative to the signal intensity of the Norm cells. Data are mean ± SE of 4-5 independent experiments. Values marked with different letters (a, b, c) are significantly different from each other (*P<0.05*), but values marked with common letters (b and bc; bc and c) are not significantly different from each other (*P>0.05*).

We analyzed the H_2_O_2_-levels in zebrafish embryos in Norm, Hypo, and Reoxy conditions using HyPer1 and OVG probes. Although the HyPer1-based analysis is suitable for capturing intracellular H_2_O_2_ changes with high sensitivity, there is a limitation in comprehensively capturing the amount of H_2_O_2_ inside and outside the cells. Therefore, we also used a chemical H_2_O_2_ probe (OVG) (**Figure 2A**). The 24 hpf zebrafish embryos were exposed to OVG for 8 hr in Hypo and Reoxy to measure fluorescence intensity. As a result, the OVG signal of Reoxy embryos was, on average, 11.5-fold stronger than that of the stage-matched Norm specimens. In addition, when comparing the OVG-treated Norm and Reoxy embryos at the same chronological age (32 hpf), the Reoxy embryos also showed a 2.9-fold stronger OVG signal than that of the Norm embryos (**Figure 2B**). This study also examined the changes in H_2_O_2_ levels produced by the Reoxy condition in mammalian cells. Namely, HEK293T cells were maintained for 14 hr in a hypoxic chamber with 5 μM OVG, and the change in OVG signal was observed after the transition to the Reoxy condition (**Figure 2C**). The results showed that OVG-derived fluorescence was very low immediately after Reoxy, but it increased 1.8-fold compared to the Norm control by 5 min after Reoxy, and it increased to more than 3-fold from that of the Norm control until 30 min after Reoxy (**Figure 2D**).

### Effect of Nox-Generated H_2_O_2_ on Catch-Up Growth

**Figure 3:**
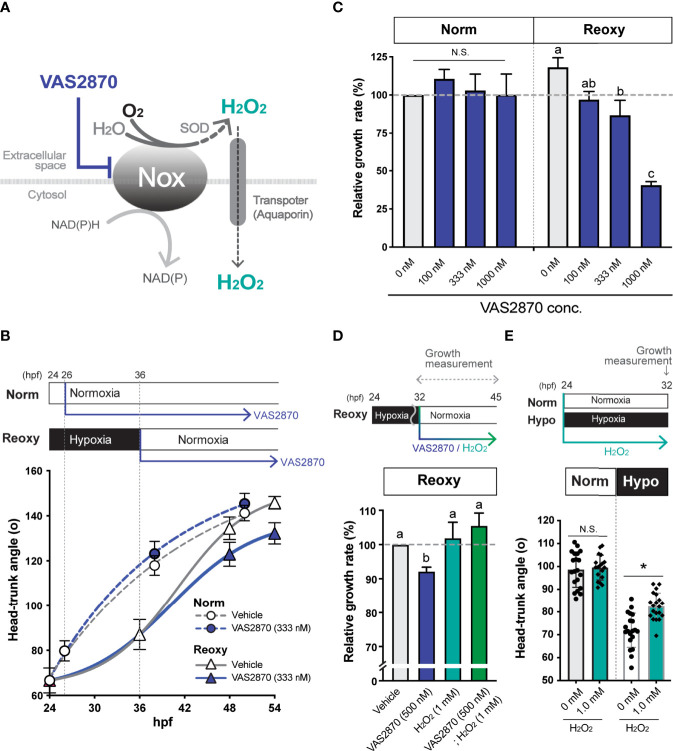
NADPH-oxidase (Nox)-generated H_2_O_2_ is crucial for the hypoxia/re-oxygenation-induced embryonic catch-up growth. **(A)** Schematic diagram of NADPH oxidase (Nox) function, which produces H_2_O_2_, a redox signaling molecule, near the plasma membrane. The Nox inhibitor, VAS2870, reduces the Nox-dependent production of H_2_O_2_ in the vicinity of the plasma membrane. Most Nox generate superoxide which is converted to the H_2_O_2_ by superoxide dismutase (SOD). **(B, C)** Effects of the Nox-inhibitor VAS2870. Wild-type zebrafish embryos were raised following the experimental regime depicted in the panel **(B)** diagram. Head-Trunk Angle (HTA) was determined at the indicated time points **(B)**. Data are average ± SD, n=9-20. **(C)** Changes in the relative growth rate of embryos in the Norm (26-38 hpf) and Reoxy (36-48 hpf) groups. The control (vehicle=0.1% DMSO) group was set as 100%. Data are mean ± SE of 3 independent experiments. Values marked with different letters (a, b, c) are significantly different from each other (*P<0.05*), but values marked with common letters (a and ab; ab and b) are not significantly different from each other (*P>0.05*). N.S. means not significantly different (P>0.05). **(D)** Changes in the relative growth rate of embryos in the Reoxy (36-48 hpf) embryos treated with or without VAS2870 and H_2_O_2_. The vehicle alone group was set as 100%. Data are mean ± SE of 3 independent experiments. Values marked with different letters (a, b) are significantly different from each other (*P<0.05*). **(E)** Changes in HTA of the Norm and Hypo embryos at 32 hpf with or without 8 hr H_2_O_2_ treatment. Data are average ± SD, n=18-20. The asterisk (*) denotes statistical difference at *P<0.05*. N.S. means not significantly different (P>0.05).

Wild-type zebrafish embryos were treated with a Nox inhibitor (VAS2870) under both Norm and Reoxy conditions (**Figure 3A**). As a result, a significant decrease in growth rate was observed in 333 nM of VAS2870-treated Reoxy embryos, but no effect was observed in Norm embryos with the same treatment (**Figure 3B**). The Reoxy-specific growth inhibition of VAS2870 was concentration-dependent (**Figure 3C**). To confirm whether this result was due to inhibition of H_2_O_2_ production by Nox, we further added H_2_O_2_ to the VAS2870 treated groups. The results showed that catch-up growth in the group treated with VAS2870 plus H_2_O_2_ was restored to the same level as the control group. Furthermore, the embryos treated with H_2_O_2_ alone resulted in the same growth rate as the control embryos (**Figure 3D**). To test if the increase in H_2_O_2_ was sufficient for growth acceleration, H_2_O_2_ was added to Norm and Hypo embryos. The results showed that the growth under the Norm condition was not accelerated by adding 1 mM H_2_O_2_, whereas exogenous H_2_O_2_ significantly accelerated growth under the Hypo condition (**Figure 3E**).

### Effect of Nox-Generated H_2_O_2_ on MAPK-Signaling

**Figure 4:**
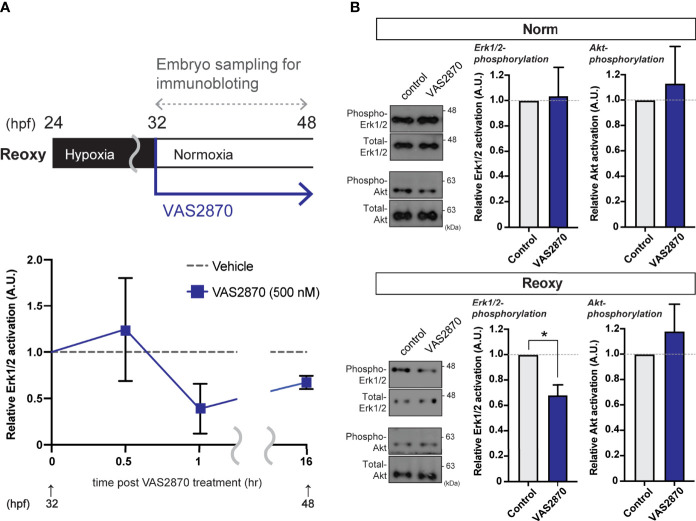
Nox-mediated generation of H_2_O_2_ is crucial for the hypoxia/re-oxygenation-induced Erk1/2-phosphorylation. **(A)** Time-course Erk1/2 phosphorylation changes of the VAS2870-treated embryos. Outline of experimental schedule is shown above the data. Reoxy embryos treated with or without VAS2870 were sampled at various timing, and the Erk1/2-phosphorylation levels were tested by immunoblotting. Relative Erk1/2-phosphorylation levels were shown as mean ± SE of 2-3 independent assays. **(B)** Erk1/2- and Akt- phosphorylation changes of the VAS2870-treated embryos. Embryos treated with or without VAS2870 (500 nM) were sampled at 48 hpf (16 hr post-re-oxygenation), and the Erk1/2- and Akt-phosphorylation levels were tested by immunoblotting. Relative Erk1/2- and Akt- phosphorylation levels were shown as mean ± SE of 2-3 independent assays. The asterisk (*) denotes statistical difference at *P<0.05*.

Cell lysate extracted from wild-type zebrafish embryos treated with VAS2870 during the Reoxy condition (from 32 hpf immediately after the start of Reoxy) was subjected to immunoblot analysis. Compared to Vehicle (DMSO) treatment, the relative ERK1/2 phosphorylation was reduced (to less than 50% of the Vehicle control) in the 1 hr-VAS2870 treatment, and the effect was observed up to 16 hr post-treatment (**Figure 4A**). However, ERK1/2 relative phosphorylation by VAS2870 was not observed in Norm embryo at 48 hpf, and VAS2870 did not reduce the Akt relative phosphorylation in both Norm and Reoxy embryos (**Figure 4B**). In addition, the H_2_O_2_ co-treatment with VAS2870 was prone to restoring the Erk1/2 phosphorylation level, which was reduced by the VAS2870 treatment alone (**Figure S1**).

### Identification and Primary Structure of Zebrafish Irs2

**Figure 5:**
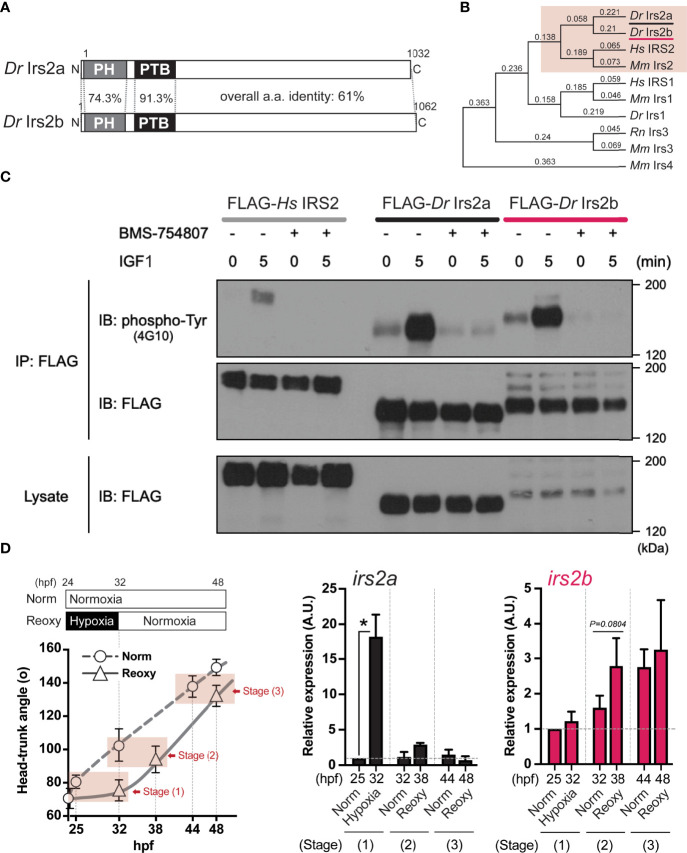
Identification, characterization, and expression of two zebrafish Irs2 genes (*irs2a/b*). **(A)** Schematic illustration of zebrafish Irs2 proteins (*Dr* Irs2a/2b). Characteristic pleckstrin homology (PH) domain and IRS-type phospho-tyrosine binding (PTB) domain are shown. **(B)** Phylogenetic analysis of the deduced amino acid sequence of Irs/IRS molecules based on the Neighbor joining method with bootstrap proportions. *Dr*, *Danio rerio*; *Hs*, *Homo sapiens*; *Mm*, *Mus musculus*; *Rn*, *Rattus norvegicus.* **(C)** Functional assay of zebrafish Irs2s in HEK293T cells. FLAG-tagged human IRS2 and zebrafish Irs2a/b were expressed, and the cells were treated with or without the specific IR/IGF-1R tyrosine kinase inhibitor BMS-754807 and were stimulated with IGF1 (100 ng/mL) for 5 minutes. Immunoprecipitation (IP) and immunoblot (IB) analyses were performed using denoted antibodies. **(D)** Real-time Q-PCR analysis of *irs2a/b* expression during zebrafish embryogenesis. Data are average ± SD, n=8-38. The sampling timing and the outline of experimental design are shown in the left. Embryos harboring statistically comparable body size was applied for the gene expression comparison: stage (1), 25 hpf Norm vs 32 hpf Hypo; stage (2) 32 hpf Norm vs 38 hpf Reoxy; stage (3), 44 hpf Norm vs 48 hpf Reoxy. Total RNA originating from whole embryos was used for cDNA synthesis, and the house-keeping gene (*β-actin)* expression was used for the internal control and normalization. The data are shown as ± SE of 3 independent assays. The Norm 25 hpf group is set as 1.0. The asterisk (*) denotes statistical difference at *P<0.05*.

In this study, we specifically explored the relationship between the Nox-generated H_2_O_2_ and the function of Irs2 in catch-up growth. First, we searched for the human IRS2 homologs in zebrafish using BLASTP with its deduced amino acid sequence. As a result, we found two molecules (Irs2a and Irs2b) that encode proteins consisting of about 1,000 amino acids with the characteristic Pleckstrin homology (PH) and phosphotyrosine-binding (PTB) domains of IRS-family proteins. The full-length amino acid sequence identity of zebrafish Irs2a and Irs2b was 61.5%, and that of the PH and PTB domains were 74.3% and 91.3%, respectively (**Figure 5A**). Phylogenetic analysis based on these primary structures showed that the zebrafish Irs2a and Irs2b were classified as members of the IRS2/Irs2 clade (**Figure 5B**).

### Functional Analysis of Zebrafish Irs2a and Irs2b

Next, the coding sequences of zebrafish Irs2a cDNA and Irs2b cDNA were cloned and expressed in HEK293T cells as a FLAG-labeled recombinant protein, and changes in tyrosine phosphorylation of the recombinant protein upon the IGF1-stimulation were examined by IP using anti-FLAG antibody followed by IB using a phospho-tyrosine antibody. The results showed that after 5 min of IGF1-stimulation, both zebrafish Irs2a and Irs2b and human IRS2 showed clear tyrosine phosphorylation. However, these IGF1-effects were no longer observed upon treatment with BMS754807, a specific inhibitor of IR/IGF1R (**Figure 5C**).

### Analysis of *irs2a* and *irs2b* Gene Expression in Normal and Catch-Up Growth

Expression levels of *irs2a* and *irs2b* genes under Hypo and Reoxy conditions were analyzed in zebrafish embryos. The results showed that *irs2a* was significantly upregulated (18.2-fold of the stage-matched control group) under the Hypo condition, but there was no apparent difference in the Reoxy condition (**Figure 5D**, *irs2a*). On the other hand, *irs2b* tended to be slightly upregulated (1.7-fold, *P*=0.0804) at a relatively early stage (38 hpf) of Reoxy compared to the stage-matched 32 hpf Norm embryos; however, in the prolonged Reoxy stage at 48 hpf, there was no apparent difference in expression compared to the stage-matched Norm embryo (**Figure 5D**, *irs2b*).

### Effects of *irs2a* and *irs2b* Translation Inhibitionon Catch-Up Growth

**Figure 6:**
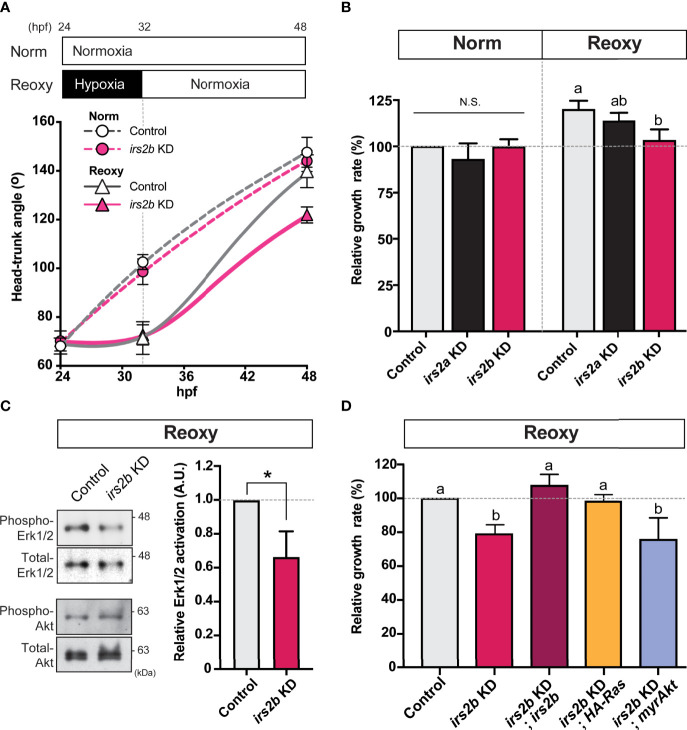
Irs2b, but not Irs2a, is required for Reoxy induced catch-up growth in the zebrafish embryo *via* its Reoxy-specific Erk1/2 activation function. **(A)** Changes in head-trunk angle. Data are average ± SD, n = 7-12. Embryos lacking the *irs2b* expression (*irs2b* KD) or control MO injected embryos were used for experiments. **(B)** Relative growth rates in Norm (26-32.5 hpf) and Reoxy (32-48.5 hpf) group. Data are mean ± SE of 3 independent experiments. Values marked with different letters (a, b) are significantly different from each other (*P<0.05*), but values marked with common letters (a and ab; ab and b) are not significantly different from each other (*P>0.05*). N.S. means not significantly different (P>0.05). **(C)** Immunoblot analysis of the phosphorylation levels of Akt and Erk1/2 under Reoxy condition (48 hpf). Data are mean ± SE of three independent experiments. The asterisk (*) denotes statistical difference at *P<0.05*. **(D)** Rescue experiments. Changes in head-trunk angle in the indicated experimental groups. Data are mean ± SE of 2-4 independent experiments. Values marked with different letters (a, b) are significantly different from each other (*P<0.05*).

Next, we observed changes in the growth rate of *irs2a/b* deficient embryos in Norm and Reoxy. As a result, loss of *irs2b* resulted in an evident growth inhibition only in the Reoxy condition, whereas there was no significant difference in growth rate in the loss of* irs2a* (**Figures 6A, B**). Furthermore, there was no significant difference in the growth rate of *irs2a* or *irs2b* deficient embryo in Norm condition. A similar comparison was made in the *irs2a/b* double-knockdown embryo. The growth rate of the *irs2a*/*b* double deficient groups resulted in almost the same growth loss as that of the *irs2b* alone knocked down embryos (**Figure S2**). Gene knockdown efficiency was confirmed using RNAs encoding fluorescent protein harboring each MO target sequence. The fluorescence was fully lost in embryos microinjected with specific MOs (**Figure S3**).

### Importance of *irs2b* on MAPK-Pathway in Re-Oxygenation Condition

In zebrafish embryos microinjected with *irs2b* MO, protein samples were prepared from embryos 48 hr after fertilization in Norm and Reoxy embryos for IB analysis. In addition, the levels of phospho- and total-Erk1/2 during the Reoxy condition were investigated. As a result, the level of phospho-Erk1/2 was significantly reduced in the *irs2b* knocked down embryos (**Figure 6C**, phospho-Erk1/2), but the activity of the PI3K-pathway (which was monitored by the Akt phosphorylation) was not significantly different in the *irs2b* knocked down embryos compared to the control embryos in Reoxy condition (**Figure 6C**, phospho-Akt; **Figure S4**). In the *irs2b* MO-injected group, catch-up growth was suppressed along with the decreased the phosphorylation levels of the Erk1/2 representing the activity of the MAPK-pathway (**Figures 6B–D**). Therefore, we performed rescue experiments using various synthetic RNAs to confirm whether the MAPK-pathway is crucial for the *irs2b* action in catch-up growth. The *irs2b* RNA, HA-RasV12, and myrAkt were microinjected into 1-2 cell stage embryos simultaneously with *irs2b* MO, and their growth rates in Reoxy condition were compared. As a result, when *irs2b* RNA or HA-RasV12 RNA was co-injected with *irs2b* MO, the growth delay caused by *irs2b* MO disappeared. However, co-injected myrAkt RNA failed to eliminate the growth-inhibitory effect of *irs2b* deficiency (**Figure 6D**). These results confirmed the implication of Irs2b-MAPK pathway but not PI3K pathway during reoxygenation-induced catch-up growth.

### Effects of H_2_O_2_ on Irs2b-Mediated Signaling and Catch-Up Growth

**Figure 7:**
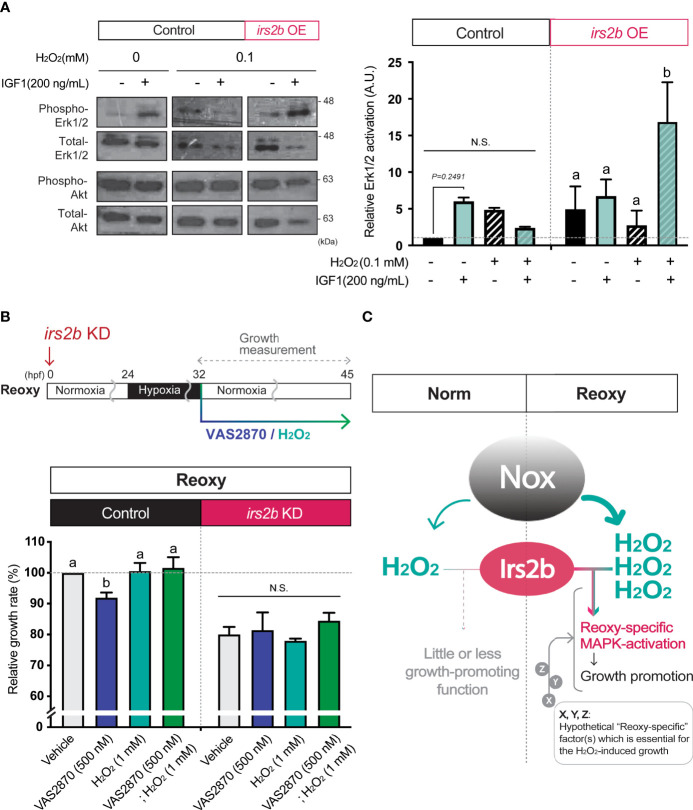
Irs2b mediates H_2_O_2_-dependent Erk1/2-phosphorylation and catch-up growth. **(A)** Immunoblot analysis of the IGF1 (200 ng/mL) -induced phosphorylation levels of Erk1/2 in the presence or absence of H_2_O_2_ and Irs2b in HEK293T cells. Cells harboring the *irs2b* overexpression (*irs2b* OE) or not (control) were used. Data are mean ± SE of 2 independent experiments. Values marked with different letters (a, b) are significantly different from each other (N.S. means not significantly different (*P<0.05*). **(B)** Changes in the relative growth rate of embryos in the Reoxy (32-45 hpf) embryos treated with or without VAS2870 and H_2_O_2_. Embryos lacking the *irs2b* expression (*irs2b* KD) or control MO injected embryos (Control) were used for experiments. The vehicle alone group was set as 100%. Data are mean ± SE of 3 independent experiments. Values marked with different letters (a, b) are significantly different from each other (*P<0.05*). **(C)** A proposed model. The Nox generates more H_2_O_2_ in Reoxy than in Norm, and the H_2_O_2_ facilitates Irs2b mediated Erk1/2 activation to induce catch-up growth. Thus the Irs2b serves as a downstream effector of re-oxygenation-induced H_2_O_2_. Since the excess H_2_O_2_ is insufficient for the significant growth acceleration in the Norm condition, other hypothetical factors (X, Y, Z, in this model) collaborating with the H_2_O_2_-Irs2b-Erk1/2 signaling would be involved in this catch-up growth model.

Since the H_2_O_2_ levels are higher in Reoxy than in Norm in culture cells, we analyzed whether changes in H_2_O_2_ levels alter the activity of the MAPK-pathway downstream of Irs2b in HEK293T cells. Overexpression of Irs2b increased basal Erk1/2-activity with or without IGF1. On the other hand, when 0.1 mM H_2_O_2_ was added, Erk1/2-activity was not enhanced by IGF1 in control cells without I rs2b overexpression. Importantly, it was significantly increased upon IGF1-stimulation in Irs2b overexpressing cells (**Figure 7A**, Erk1/2). In contrast, PI3K-pathway was not significantly altered by IGF1 in *Irs2b* overexpressing cells within the current 15 min stimulation, regardless of the H_2_O_2_ treatment (**Figure 7A**, Akt). To determine if H_2_O_2_ is associated with *irs2b* function in Reoxy, *irs2b* deficient embryos were treated with VAS2870 and H_2_O_2_ at the beginning of Reoxy. In the Reoxy-induced catch-up growth, a decrease in growth rate was observed in VAS2870-treated control MO-injected embryos, but the growth rate was recovered to the normal level by the H_2_O_2_ co-treatment with VAS2870. On the other hand, the growth rate of *irs2b* deficient embryos was lower in the vehicle control MO-injected embryos. Notably, the growth rate change induced by VAS2870 and H_2_O_2_ was utterly abolished in the *irs2b* deficient embryos (**Figure 7B**).

## Discussion

Immediately after the transition from Norm to Hypo, the H_2_O_2_ levels in zebrafish embryos markedly decreased, and it increased immediately after the transition from Hypo to Reoxy. It is noteworthy that these changes in H_2_O_2_ levels occurred within a brief period (approximately 10-15 min post environmental change). Such dynamic changes in H_2_O_2_ levels are likely to occur without gene expression. Because oxygen is often consumed for the formation of H_2_O_2_, it makes sense that the levels of newly synthesized H_2_O_2_ drop under the Hypo condition. Nevertheless, monitoring the* in vivo *H_2_O_2_ levels in living embryos is always challenging. Current data would be an important demonstration that the H_2_O_2_ levels decreases in living embryos in response to scary environmental oxygen. Furthermore, the amount of H_2_O_2_ was higher in Reoxy than in Norm. This supports the notion that the internal physiological condition is different between Norm and Reoxy, even though the two groups have the same environmental oxygen concentration. It was shown that, in ischemia/reperfusion, the H_2_O_2_ levels increased (20). In the present model, systemically elevated H_2_O_2_ was also confirmed in embryos (as well as in culture cells) under the Reoxy, suggesting that the H_2_O_2_ augmentation is a conserved cellular response to the Reoxy condition. We know that intracellular embryonic growth signaling (e.g., IGF-signaling) in Reoxy is distinct from Norm (2, 17), leading to catch-up growth. Given that H_2_O_2_ is prone to modify intracellular signal mediator proteins, it is conceivable that H_2_O_2_ alters somatotropic signaling, which controls the catch-up phenomenon in Reoxy.

Since the cell membrane is an ignition site of growth factor signaling, H_2_O_2_ produced in the vicinity of the cell membrane could have roles which cannot be ignored in catch-up growth. Because Nox is a vital membrane resident H_2_O_2_-generator in the zebrafish embryo (34), and because hypoxia is known to induce some *nox* gene expression and to enhance Nox function (35, 36), we focused on the role of Nox in Reoxy-induced catch-up growth in this study. Inhibition of Nox by the VAS2870 significantly reduced the growth rate and MAPK-signaling in Reoxy but not in Norm. In addition to H_2_O_2_, Nox is also known to produce NADP^+^ (37). The addition of exogenous H_2_O_2_ restored the catch-up growth and MAPK-pathway activation in the presence of the Nox inhibitor. Therefore, it was highly likely that the Nox-generated H_2_O_2_ at the cell membrane was necessary for catch-up growth in the current model. The detailed molecular mechanism for this Reoxy-specific H_2_O_2_ generation is still unclear. We failed to find any significant increase of relative gene expression for the *nox* family and its regulators in neither Hypo nor Reoxy; some of the gene expressions were even lower in Hypo and Reoxy compared to their stage-matched Norm embryo at the whole body level (**Figure S5**). Since related studies have shown that *nox* is expressed in blood cells and particular tissue such as the brain, analysis focusing on the tissue/cell type-specific *nox* expression is needed in the future. Alternatively, Reoxy-specific regulation of Nox activity is crucial, though we did not test in this work. We also found the ubiquitous expression pattern of *irs2b* in the zebrafish embryo, though some of the Norm fish had lower expression in mid trunk region and some of the Reoxy embryo had more vital global expression (**Figure S6**). Another crucial result is that the growth-promoting effect of H_2_O_2_ is observed in Reoxy and Hypo, but not in Norm (**Figure 3E**). Similarly, we tried to increase the H_2_O_2_ levels by the loss of the H_2_O_2_-eliminating enzyme gene, *catalase* (38). The *catalase*-mutant is a null mutation, and this gene is functional as the overexpression of this gene reduced the levels of H_2_O_2_ *in vivo* (38). As results, the *catalase *mutant did not enhance the growth rate in Norm (**Figure S7**). These results, in conjunction with the fact that the addition of H_2_O_2_ *per se* did not significantly increase the growth rate in Norm embryos, indicate that the increased H_2_O_2_ levels are necessary but insufficient for growth acceleration found in Reoxy. It is plausible that the prolonged hypoxia changes gene expression and such factors potentially collaborate with H_2_O_2_ to accelerate growth, especially in the beginning of Reoxy. Indeed, redox regulators and signal modulators (such as *gpx3*, *txnipa*, *irs2a*, *mknk2b*, and *arrdc3b*) were upregulated in Hypo embryos compared to stage-matched Norm embryos in RNA-sequencing analysis (Data not shown). In addition, increased H_2_O_2_ levels affect the activities of protein phosphatases (39). Given the prominent roles of phosphatases in the insulin/IGF-signaling (40), it is plausible that H_2_O_2_ diminishes the phosphatase activity in Reoxy, thereby the unique growth-promoting role of Irs2b found in this model. Therefore, the investigation focused on modifying phosphatase activity seems likely to link to the currently revealed Irs2b mode in Reoxy. The identification of hypothetical “Reoxy-specific” factor(s), which is essential for the H_2_O_2_-induced growth acceleration (designated as X, Y, Z in **Figure 7C**), would also await future investigation.

This study focused on *irs2* genes as effectors of Nox-generated H_2_O_2_ to control IGF/Igf-MAPK signaling because the Irs2 showed altered function in response to the increased H_2_O_2_ (25, 26), anti-inflammatory function (41), and some studies reported a biased IGF-MAPK signaling in culture cells (42, 43). Thus, we characterized zebrafish *irs2* genes (*irs2a*/*irs2b*) and found that both genes encode functional Irs2 protein showing efficient tyrosine phosphorylation upon the IGF-stimulation in culture cells. The tyrosine phosphorylation of the IRS is known for inevitable events for recruiting the PI3K-p85 regulatory subunit and Grb2/SOS MAPK-mediator to the complex/membrane region that initiates signal transductions of both PI3K- and MAPK-pathways (8). Expression analysis revealed that the *irs2a* expression markedly gained under Hypo condition; however, the loss of *irs2a* did not resulted in the significant loss of the growth in any conditions. These data suggest that the *irs2a* gene is not decently involved in the growth in this model organism, though the loss of the *irs2a* slightly reduced the growth in Reoxy. The loss of *irs2a* prone to reduce Akt phosphorylation in Reoxy (data not shown), which may lead a minor growth deficit (**Figure 6B**). The loss of *irs2a* or *irs2b* tended to increase Akt phosphorylation in Norm (data not shown). Since the IRS2/Irs2 generally activates Akt signaling, the current data puzzles. Also, since the Irs2-Akt signaling plays a critical role in proper nutrient and energy metabolism in mice model, the loss of *irs2a* and *irs2b* may lead to the phenotypically invisible metabolic defects. The Irs1 in mice also have metabolic roles, which was not characterized in the zebrafish model. The metabolic role of *irs* genes in the fish model is an another important research subject in the future. About the Irs2 function, the most important finding in the current study was that growth rate and MAPK-pathway were significantly reduced by the loss of the *irs2b *in Reoxy embryos. The *irs2b* function in the MAPK-activation was not found in Norm (**Figure S8**). In addition, forced expression of *irs2b* RNA or HA-RasV12 (an upstream factor of the MAPK-pathway) restored catch-up growth in *irs2b *deficient embryos, but the myrAkt expression failed. These results imply that *irs2b* specifically increases input from the Igf1r to the MAPK-pathway during the Reoxy condition to facilitate catch-up growth. This study would provide new insights into the function of IRS2/Irs2 in body growth; it partially proposes a molecular basis for the previously unsolved question of why the Igf1r-MAPK (but not the Igf1r-PI3K) has a biased augmentation in Reoxy in the zebrafish model. The molecular basis explaining why the *irs2b* is involved to the growth more than the *irs2a* and other signaling mediators is currently unknown, but we speculated that the posttranslational modifications specific to the Irs2b is likely involved the efficient MAPK activation in Reoxy condition. Future studies are needed to uncover this part.

The proper ROS generations during embryonic growth and development and the Nox molecule(s) play crucial roles in the neurite outgrowth of retinotectal connections and optic tectum development in zebrafish embryos in Norm embryos (44, 45). In response to adverse conditions such as wounds, Nox-induced H_2_O_2_ is an initiation cue for the healing process in the damaged tissue (46). This time, we found that the Nox-inhibitor significantly blunted the growth in the Reoxy condition, and the addition of H_2_O_2_ restored it. The Reoxy-specific Nox action for body growth is an essential finding of the current study. Notably, the growth rate of *irs2b* deficient embryo was not further reduced by VAS2870. Furthermore, the addition of H_2_O_2_ did not restore the growth of these Nox-inhibited *irs2b* deficient embryos. These results strongly suggest that H_2_O_2_ cooperatively functions with *irs2b*. In fact, in mammalian cells overexpressing Irs2b, IGF1-stimulation in the presence of H_2_O_2_ significantly increased the Erk1/2 phosphorylation (**Figure 7A**). Since these results were not observed in cells without exogenous H_2_O_2_-supplementation, it was strongly inferred that the function of Irs2b for efficient transduction of IGF1-stimulus to the MAPK-pathway occurs only when a certain amount of H_2_O_2_ and Irs2b are present simultaneously. The HEK293T cells should express endogenous human IRS2 at a certain level, but the control experiment without no exogenous Irs2b expression failed to show the H_2_O_2_-induced significant augmentation of Erk1/2 phosphorylation upon the IGF-stimulus. These data suggest either that the Irs2b and human IRS2 have a distinct function under the effects of H_2_O_2_ or that the H_2_O_2_-mediated IRS2/Irs2b function requires a certain expression level. Earlier studies showed that the Nox4-derived H_2_O_2_ facilitated the sustained IGF-action *via* Src-oxidation in vascular smooth muscle cells under hyperglycemic conditions (27). The *nox4* gene expression was too low to detect in this study, and the Irs2b may play a role with oxidized-Src induced by other Noxes, or the Irs2b itself could be oxidized by the Nox-produced H_2_O_2_ to change its function. It has been shown that H_2_O_2_ can change the structure and function of proteins through oxidative modification (47). Another idea is that Irs2b may alter its function only under catabolic conditions such as hypoxia. It is widely accepted that some of the proteins (including deacetylases such as Sirtuins, Sirt1-7) are highly active in the catabolism condition (48), and maybe its activity remains higher for a while at the removal of catabolic condition. The Sirt1 and IRS2 form a complex to activate IGF-MAPK-pathway when the neuron was exposed to H_2_O_2_ (43). Recent reports have shown that Sirt1 contributes to catch-up growth in mice models (49).

In this study, we found that the levels of H_2_O_2_ are increased under the Reoxy condition, and the Nox-dependent H_2_O_2_ is indispensable for the MAPK-activation and catch-up growth in the zebrafish embryo. We also identified *irs2b* as a gene required for the Nox-mediated catch-up growth, where the Irs2b functions as an effector of Nox-generated H_2_O_2_ to maintain the Reoxy-specific Igf-MAPK-pathway and body growth. Thus, these molecules control Reoxy-induced catch-up growth in the zebrafish embryo. Though endogenous early developmental H_2_O_2_ production is necessary for normal neurogenesis and tissue regeneration (22, 23), this study reveals a novel Reoxy-specific physiological function of the Nox-generated H_2_O_2_ in systemic growth. Another important idea from the current study is the context-dependent (Reoxy-specific) growth-promoting role of the Irs2b. So far, studies in mice have reported that *Irs1* is essential for body growth (11), but *Irs2* contributed to normal body growth only a little (13). On the other hand, recent studies have shown that *Irs2* is required for normal body growth in rats (14) and that Irs2 protein potentially plays a role in the growth-promoting mechanism with some specific interacting molecule(s) (50). Irs2 is crucial for various physiological functions, including maintaining energy homeostasis (12, 51), and the Erk1/2 contributes to various cell-fate determinations such as proliferation and differentiation (52), the elucidation of the H_2_O_2_-induced Irs2b-MAPK function should be a crucial research subject. Future biochemical characterization of the detailed Irs2b modification caused by the membranous H_2_O_2_ in Reoxy would lead to a new avenue for a better understanding of how the redox signaling generates context-dependent growth signaling.

## Data Availability Statement

The original contributions presented in the study are included in the article/**Supplementary Material**. Further inquiries can be directed to the corresponding author.

## Ethics Statement

The animal study was reviewed and approved by Care and Use of Laboratory Animals prepared by Kanazawa University; Center for Interdisciplinary Research in Biology, Collège de France.

## Author Contributions

HK, MV, SV and CR conceived and designed these experiments. AZ, FHi, MT, YY, YK and HK performed the experiments. AZ, FHi, MT, YY, CR, and HK analyzed the data. HK, YK, FHa, S-IT, SV contributed reagents and materials/analysis tools. AZ, FHa, MV, S-IT, SV, CR and HK wrote/edit the paper. All authors contributed to the article and approved the submitted version.

## Funding

This work was supported by JSPS-MEAE-MESRI (JAPAN-France) collaboration SAKURA program [JPJSBP120193208] to HK and SV. This work was also partially supported by the Japan Society for the Promotion of Science, Grant-in-Aid for Young Scientists (B) [15K18799] and Grant-in-Aid for Scientific Research (C) [18K06014] to HK. Invitation program from Université Paris Cité.

## Conflict of Interest

The authors declare that the research was conducted in the absence of any commercial or financial relationships that could be construed as a potential conflict of interest.

## Publisher’s Note

All claims expressed in this article are solely those of the authors and do not necessarily represent those of their affiliated organizations, or those of the publisher, the editors and the reviewers. Any product that may be evaluated in this article, or claim that may be made by its manufacturer, is not guaranteed or endorsed by the publisher.

## Abbreviations

PI3K, phosphatidylinositol 3-kinases; MAPK, mitogen-activated protein kinase; Erk1/2, extracellular signal−regulated protein kinase 1/2; CDS, coding sequence; eGFP, enhanced green fluorescent protein; HA-RasV12, a type of constitutive active Ras family of oncogenic protein; myrAkt, a myristoylated constitutive active Akt1 protein; Grb2, growth factor receptor-bound protein 2; SOS, son of sevenless Ras/Rac guanine nucleotide exchange factor 1; *gpx3*, glutathione peroxidase 3 gene; *txnipa*, thioredoxin interacting protein a gene; *mknk2*, MAPK Interacting Serine/Threonine kinase 2 gene; *arrdc3b*, arrestin domain containing 3b; Src, Proto-oncogene tyrosine-protein kinase Src.
