# Supplementary material for: NADPH-Oxidase Derived Hydrogen Peroxide and Irs2b Facilitate Re-oxygenation-Induced Catch-Up Growth in Zebrafish Embryo
Source: Front Endocrinol (Lausanne). 2022 Jul 1;13:929668. doi: 10.3389/fendo.2022.929668 (PMC9283716; doi:10.3389/fendo.2022.929668)
Supplement: Supplementary file 1 [file DataSheet_1.pdf]

Supplemental materials:

NADPH-oxidase derived hydrogen peroxide and Irs2b facilitate re-oxygenation-induced catch-up growth in zebrafish embryo.

Ayaka Zasu[1], Futa Hishima[1], Marion Thauvin[2,3], Yosuke Yoneyama[4,5], Yoichiro Kitani[6], Fumihiko Hakuno[4], Michel Volovitch[2,7,8], Shin-Ichiro Takahashi[4], Sophie Vriz[2,8,9], Christine Rampon[2,8,9] and Hiroyasu Kamei[1§]

1. Faculty of Biological Science and Technology, Institute of Science and Engineering, Kanazawa University, Noto, Ishikawa, Japan
2. Center for Interdisciplinary Research in Biology (CIRB), Collège de France, CNRS, INSERM, PSL Research University, Paris, France
3. Sorbonne Université, Paris, France
4. Departments of Animal Sciences and Applied Biological Chemistry, Graduate School of Agriculture and Life Sciences, The University of Tokyo, Tokyo, Japan
5. Institute of Research, Tokyo Medical and Dental University, Tokyo, Japan
6. Noto Marine Laboratory, Division of Marine Environmental Studies, Institute of Nature and Environmental Technology, Kanazawa University, Noto, Ishikawa, Japan
7. Department of Biology, École Normale Supérieure, PSL Research University, Paris, France
8. Laboratoire de BioMolécules (LBM), Département de Chimie, Sorbonne Université, École Normale Supérieure, PSL University, Sorbonne Université, CNRS, 75005, Paris, France.
9. Université Paris-Cité, Faculty of Sciences, Paris, France

Legends for Supplemental Figures

Figure S1. Effects of Nox and H_2O_2 on Erk1/2 phosphorylation. Changes of the Erk1/2 phosphorylation in the VAS2870-treated embryos. Representative immunoblotting data of 1 hr post-re-oxygenation. Outline of experimental schedule is the same as shown in Figure 4A. Data are mean ± SE of 2 independent assays. The same letter denotes statistically comparable ($P>0.05$).

Figure S2. Effect of double deficiency of *irs2a/b* on embryonic growth in re-oxygenation condition. Relative growth rates in Reoxy (32-48 hpf). The control is set as 100. Data are mean ± SE of 2-6 independent experiments. Values marked with different letters (a, b) are significantly different from each other ($P<0.05$), but values marked with common letters (a and ab; ab and b) are not significantly different from each other ($P>0.05$).

Figure S3. Validation of gene knockdown. (A) Schema of the MO-target sequence and the synthetic RNA sequences. (B) To examine the efficacy of *irs2a/b* knockdown, the effects of translation block antisense MO targeting either zebrafish *irs2a* mRNA or *irs2b* mRNA were analyzed by a co-injection of synthetic Venus RNAs harboring the MO-target sequence.

Figure S4. Effect of *irs2b* deficiency on the Akt phosphorylation. Phosphorylation levels of Akt under Reoxy condition (48 hpf). Data are mean ± SE of three independent experiments. N.S. denotes statistically comparable ($P>0.05$).

Figure S5. Real-time Q-PCR (qRT-PCR) analysis of *nox* and related genes. Schematic cartoon of the sampling schedule is in the left-top panel. Total RNA originating from whole embryos was used for cDNA synthesis, and the house-keeping gene (*ef1a)* expression was used for the internal control and normalization. Primers used for this qRT-PCR analysis are listed in the Table S1 (No. 17-40). The data are shown as mean ± SE of 3-6 independent assays. Values marked with different letters (a, b, c) are significantly different from each other ($P<0.05$), but values marked with common letters (a and ab; ab and b ; b and bc; bc and c) are not significantly different from each other ($P>0.05$).

Figure S6. Spatial expression patterns of zebrafish *irs2b*. Whole-mount *in situ* hybridization analysis of *irs2b* expression in Norm and Reoxy embryos. Approximately stage-matched embryos (Norm, 44 hpf; Reoxy, 48 hpf) are subjected to the analysis. Pictures are taken at lateral view. Scale bar = 500 µm. Signals are indicated by dotted lines and arrowheads. Inset number shows penetrance of each representative staining results obtained when 10 embryos were subjected to the staining. Detailed method for the whole-mount *in situ* hybridization was followed by previous report (10). For the probe synthesis, the *irs2b* cDNA

was cloned by using primer set listed in Table S1 (No. 41-42). The cloned cDNA was inserted in pGEM-T Easy vector, then the antisense probe was prepared using the vector.

Figure S7. Effect of *catalase* deficiency on catch-up growth. (A) Changes in head-trunk angle. Data are average ± SD, n = 10-52. Embryos lacking the *catalase* expression (Cat-MUT) or control wild-type (Cat-WT) embryos were used for experiments. (B) Relative growth rates in Norm (26-48 hpf) and Reoxy (32-48 hpf) group.

Figure S8. Changes of Erk1/2 and Akt signaling in *irs2b* deficient embryos under Norm condition. Immunoblot analysis of the phospho- and total-Erk1/2 and Akt under Norm condition (48 hpf). Data are mean ± SE of 3 independent experiments. N.S. denotes statistically comparable ($P>0.05$).

Table S1. Sequence information of primers used for cDNA amplification

Primer No.	Primer name	Sequence	Purpose
1	zf Irs2a EcoRI-KZK-F	5'-TTTTGAATTCCCACCATGGCAAGTCCGCCTCTTAAAG-3'	CDS cloning for mammalian expression
2	zf Irs2a XhoI-R	5'-GAGGCTCGAGTCAATCTTGTACAGTGGTTGCAGTT-3'	CDS cloning for mammalian expression
3	zf Irs2b EcoRI-KZK-F	5'-TTTTGAATTCCCACCATGAGAATGGCGAGTCCGCCGCCGAC-3'	CDS cloning for mammalian expression
4	zf Irs2b XhoI-R	5'-GAGGCTCGAGTTAGTCTTTTACTGTTGTTGCCGTGT-3'	CDS cloning for mammalian expression
5	zf Irs2a F1	5'-CTCCGAGGTGGCATCAGTTAC-3'	RT-Q-PCR
6	zf Irs2a R1	5'-CCCCTTCACTTGCAGTCCGTATT-3'	RT-Q-PCR
7	zf Irs2b F1	5'-CCGAGAACGCATCGGTGATTT-3'	RT-Q-PCR
8	zf Irs2b R1	5'-CCTCGCTCGCTGTGCGGATCCT-3'	RT-Q-PCR
9	zf β-actin F	5'-CACGAGACCACCTTCAACT-3'	RT-Q-PCR
10	zf β-actin R	5'-ATCCAGACGGAGTATTTGC-3'	RT-Q-PCR
11	zf Irs2a-MO target-F	5'-TCGAATTCCGCCACCGATAAGTTTTGGAAACTGAG-3'	MO-Target RNA synthesis
12	zf Irs2a-MO target-R	5'-CGCCCTTGCTCACCACCCCTTTAAGAGGCGGACTT-3'	MO-Target RNA synthesis
13	zf Irs2b-MO target-F	5'-TCGAATTCCGCCACCTTCACAGCATATGAGAATGG-3'	MO-Target RNA synthesis
14	zf Irs2b-MO target-R	5'-CTCGCCCTTGCTCACATAGCCGCATTTCTTAACGT-3'	MO-Target RNA synthesis
15	pCS2 Venus vector-F	5'-GTGAGCAAGGGCGAGGAGCTGTTCA-3'	MO-Target RNA synthesis
16	pCS2 Venus vector-R	5'-GGTGGCGGAATTCGAATCGATGGGA-3'	MO-Target RNA synthesis
17	zf Nox1 F	5'-GGCCGAACTGGGATAAAGAATTTGAAC-3'	RT-Q-PCR
18	zf Nox1 R	5'-CTTGGTTCTTCGGGGATCAACATCAG-3'	RT-Q-PCR
19	zf Nox2 F	5'-GATAAGACTGCGGTGCTGGATGC-3'	RT-Q-PCR
20	zf Nox2 R	5'-ATACCAGCACCAACCAGCATCACC-3'	RT-Q-PCR
21	zf Nox5 F	5'-ATCAGATCGTGGGTTACGCC-3'	RT-Q-PCR
22	zf Nox5 R	5'-TACGCCAGTAATGGAGGCTG-3'	RT-Q-PCR
23	zf Duox1 F	5'-CCTGGGAGGACTTTCACTTTC-3'	RT-Q-PCR
24	zf Duox1 F	5'-CTTGTGCTGTCTGCCTAGTT-3'	RT-Q-PCR
25	zf Duox2 F	5'-GCTTTAGTTCTCGCCTGTGG-3'	RT-Q-PCR
26	zf Duox2 R	5'-TCACCGATCTGCCACGTATT-3'	RT-Q-PCR
27	zf Noxa F	5'-CAAACCGCTGTAGTAGAGTGC-3'	RT-Q-PCR
28	zf Noxa R	5'-ATCGCTTCCCCTCCATTTGTA-3'	RT-Q-PCR
29	zf Noxo1a F	5'-GTGTTTTCTATGTGGCAACCAAAGC-3'	RT-Q-PCR
30	zf Noxo1a R	5'-GTATCCCGCCTTTCGGTTGT-3'	RT-Q-PCR
31	zf Noxo1b F	5'-TGTCTCTGTACTGCGCGACA-3'	RT-Q-PCR
32	zf Noxo1b R	5'-GGCACGTAACCAGCCGTATTG-3'	RT-Q-PCR
33	zf p22phox F	5'-ACCTCTCTGCAGCTATTCATGG-3'	RT-Q-PCR
34	zf p22phox R	5'-TTTACGGCGCAGTTCAGGG-3'	RT-Q-PCR
35	zf p40phox F	5'-CGGTTGCTTTGTCTGCCTACTT-3'	RT-Q-PCR
36	zf p40phox R	5'-GAAGGTCTGTTTTGGGCTTCAC-3'	RT-Q-PCR
37	zf p67phox F	5'-CTGGATGCCATTCTGAAACATAAGCTG-3'	RT-Q-PCR
38	zf p67phox R	5'-GATGGAACATTGTCTATTTGAGGCTG-3'	RT-Q-PCR
39	zf Ef1a F	5'-ACCGGCCATCTGATCTACAA -3'	RT-Q-PCR
40	zf Ef1a R	5'-CAATGGTGATACCACGCTCA -3'	RT-Q-PCR
41	zf Irs2b-probe F	5'-CGCGGGAATTCGATTAGGAAGATGAGAGGTACCAG-3'	cRNA synthesis for *in situ* -hybridization
42	zf Irs2b-probe R	5'-AATTCACTAGTGATTTCTGTGTCTGAGTCTTCGTT-3'	cRNA synthesis for *in situ* -hybridization

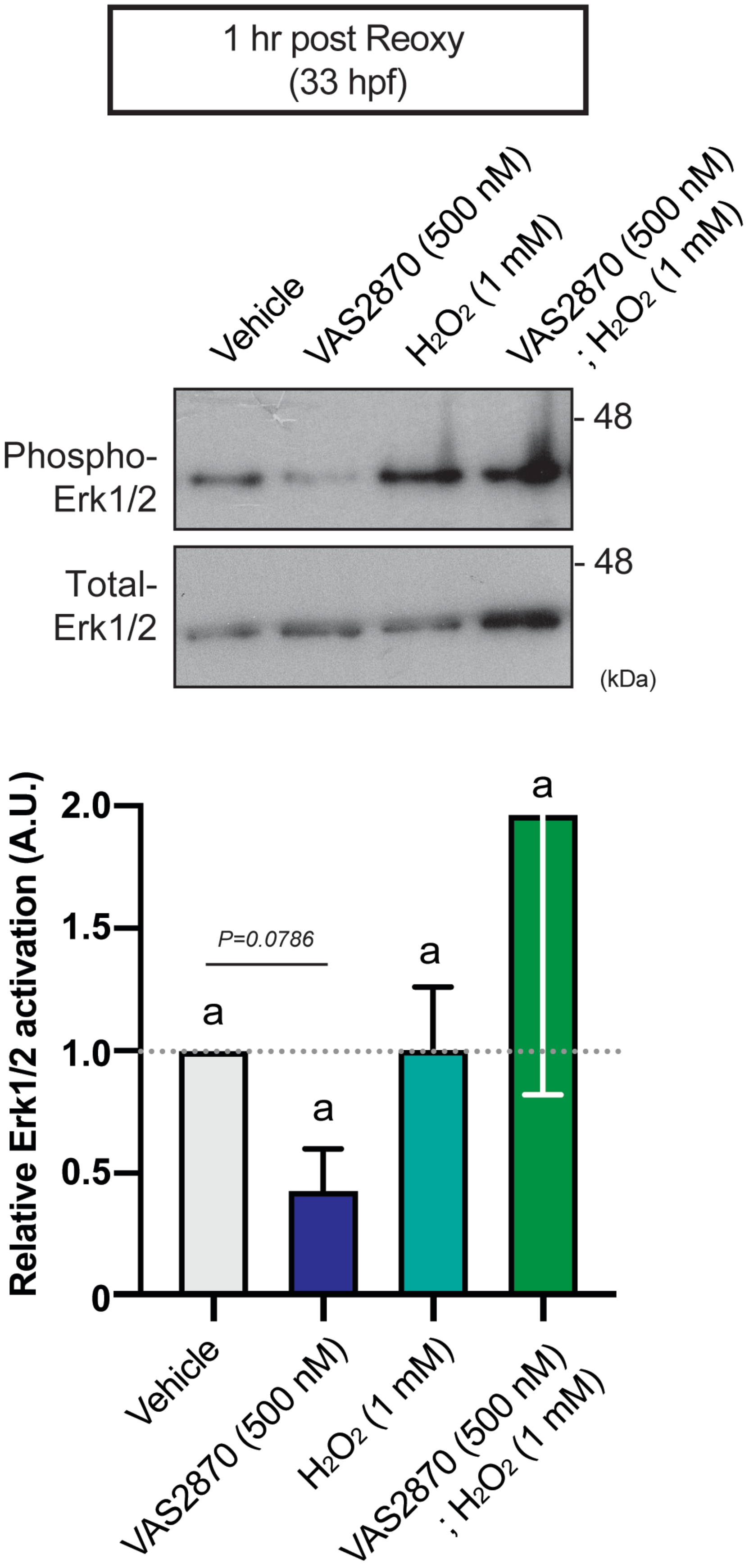
1 hr post Reoxy
(33 hpf)
Vehicle
VAS2870 (500 nM)
H2O2 (1 mM)
VAS2870 (500 nM)
; H2O2 (1 mM)
Phospho-
Erk1/2
Total-
Erk1/2
- 48
- 48
(kDa)
Relative Erk1/2 activation (A.U.)
2.0
1.5
1.0
0.5
0
P=0.0786
a
a
a
a
Vehicle
VAS2870 (500 nM)
H2O2 (1 mM)
VAS2870 (500 nM)
; H2O2 (1 mM)

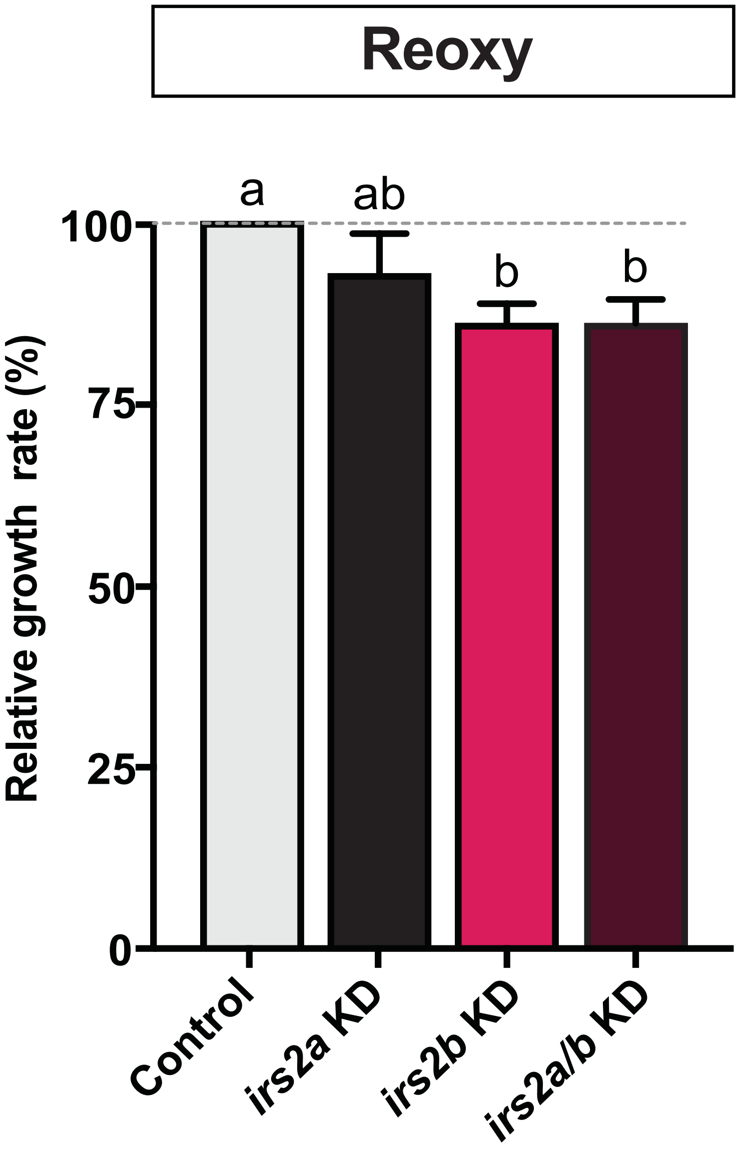
Reoxy
Relative growth rate (%)
100
75
50
25
0
a
ab
b
b
Control
irs2a KD
irs2b KD
irs2a/b KD

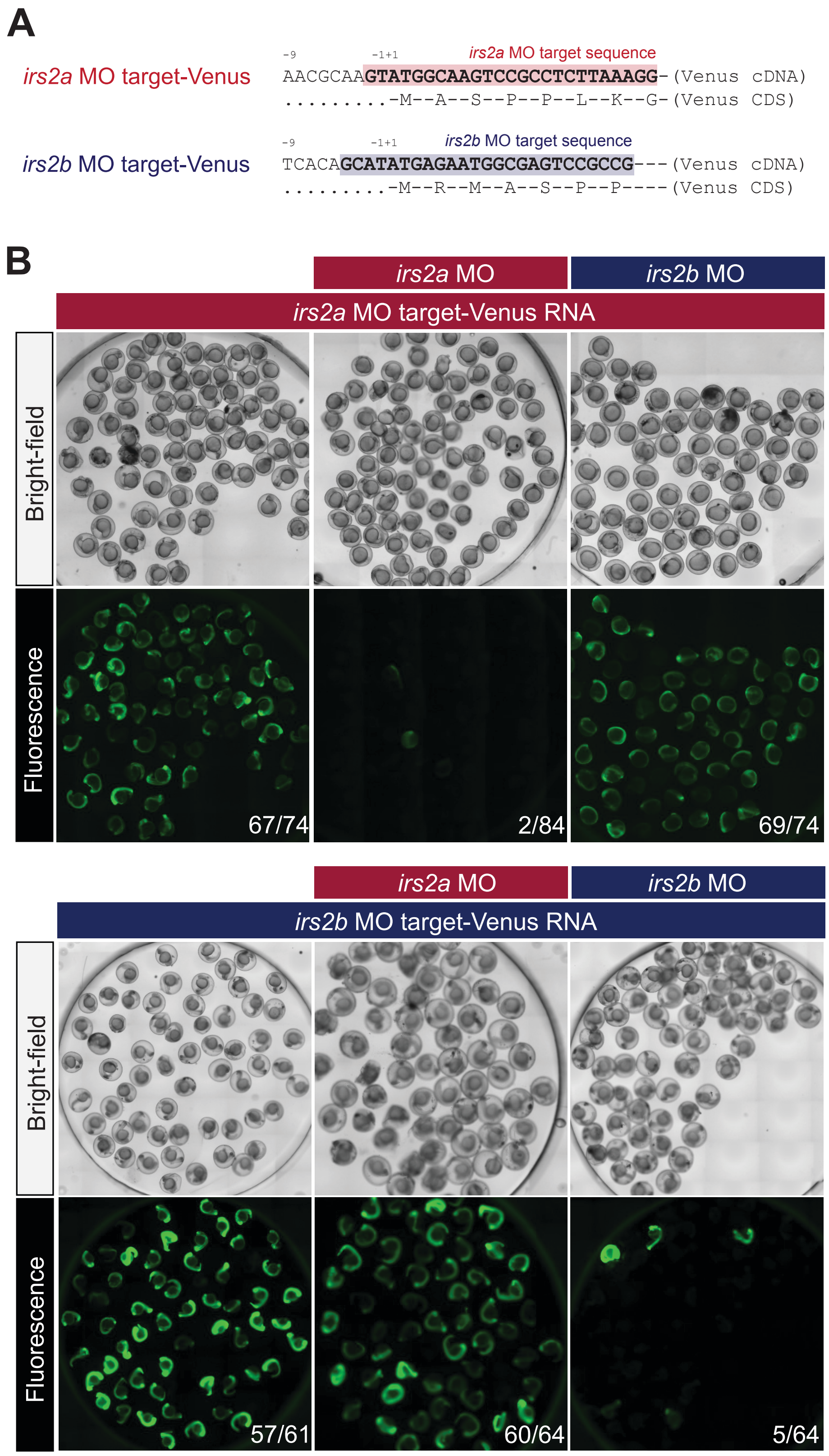
A
irs2a MO target-Venus
-9
-1+1
irs2a MO target sequence
AACGCAAGTATGGCAAGTCCGCCTCTTAAAGG-(Venus cDNA)
.........-M--A--S--P--P--L--K--G-(Venus CDS)
irs2b MO target-Venus
-9
-1+1
irs2b MO target sequence
TCACAGCATATGAGAATGGCGAGTCCGCCG---(Venus cDNA)
.........-M--R--M--A--S--P--P----(Venus CDS)
B
irs2a MO
irs2b MO
irs2a MO target-Venus RNA
Bright-field
Fluorescence
67/74
2/84
69/74
irs2a MO
irs2b MO
irs2b MO target-Venus RNA
Bright-field
Fluorescence
57/61
60/64
5/64

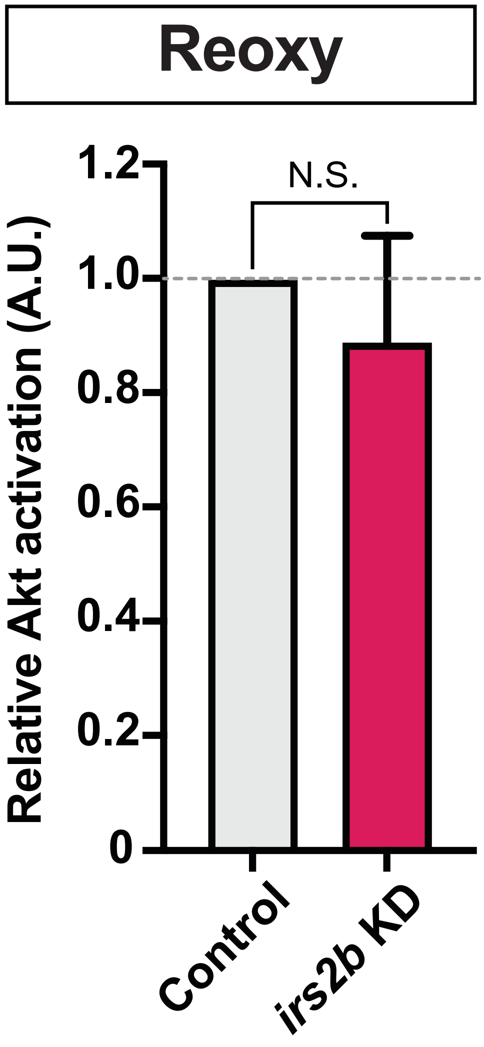
Reoxy
1.2
1.0
0.8
0.6
0.4
0.2
0
Relative Akt activation (A.U.)
N.S.
Control
irs2b KD

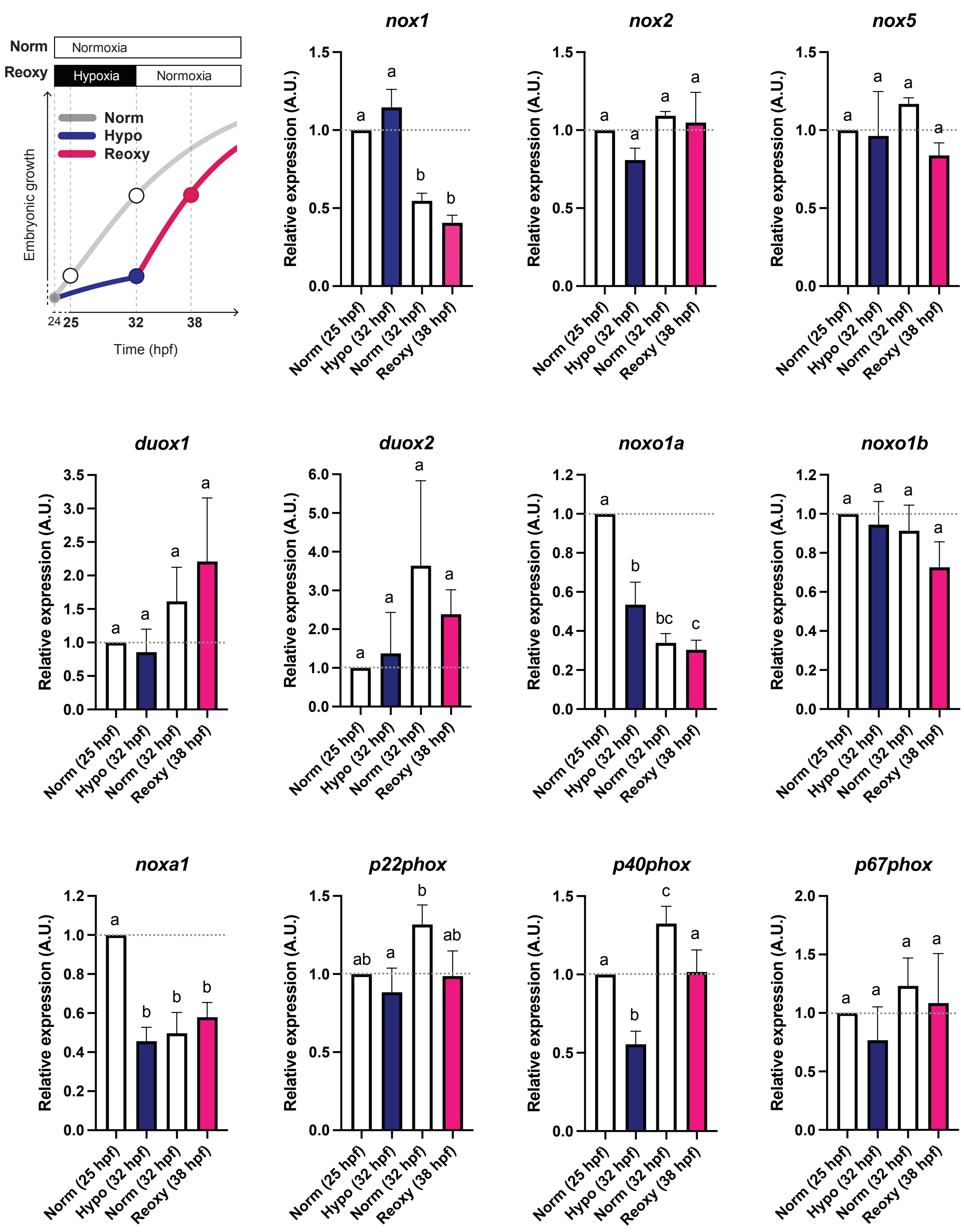
Norm
Normoxia
Reoxy
Hypoxia
Normoxia
Embryonic growth
Norm
Hypo
Reoxy
24 25 32 38
Time (hpf)
nox1
nox2
nox5
duox1
duox2
noxo1a
noxo1b
noxa1
p22phox
p40phox
p67phox
Relative expression (A.U.)
Norm (25 hpf)
Hypo (32 hpf)
Norm (32 hpf)
Reoxy (38 hpf)

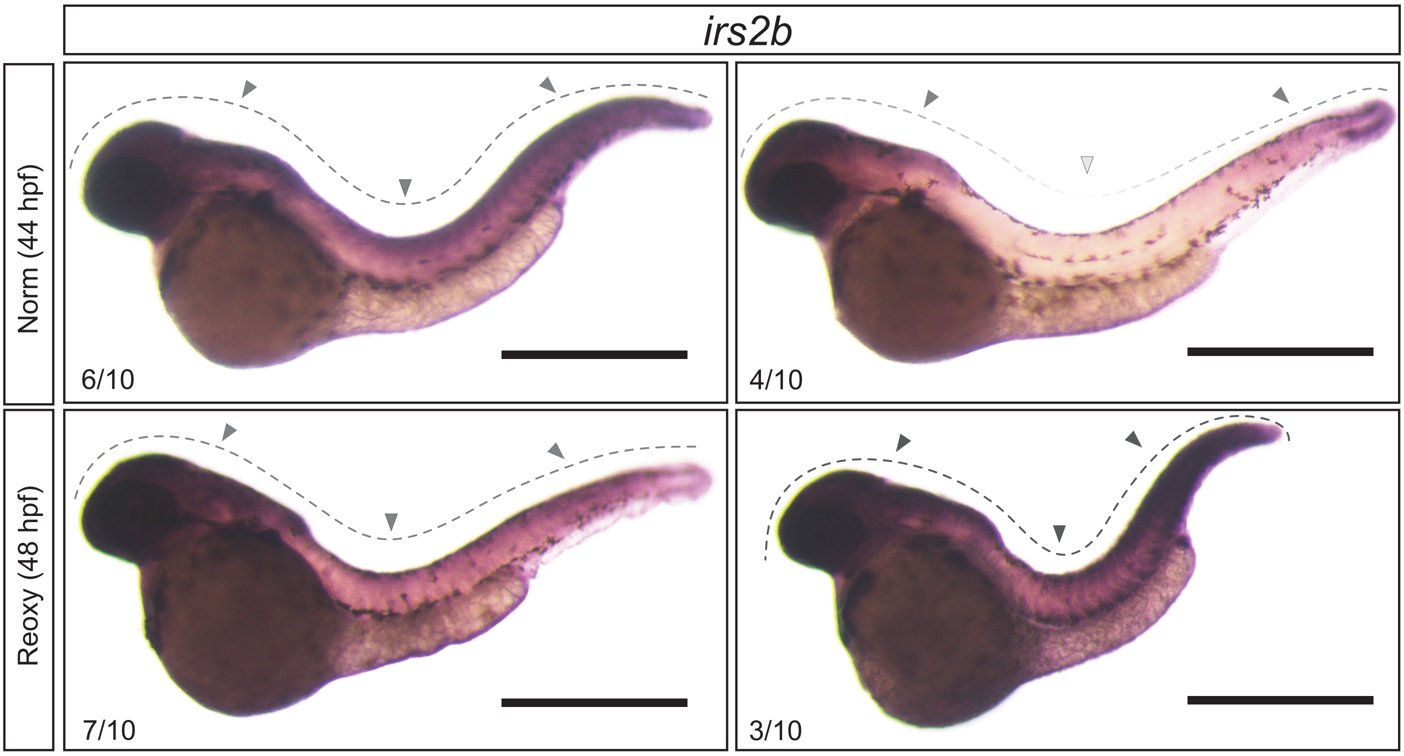
irs2b
Norm (44 hpf)
Reoxy (48 hpf)
6/10
4/10
7/10
3/10

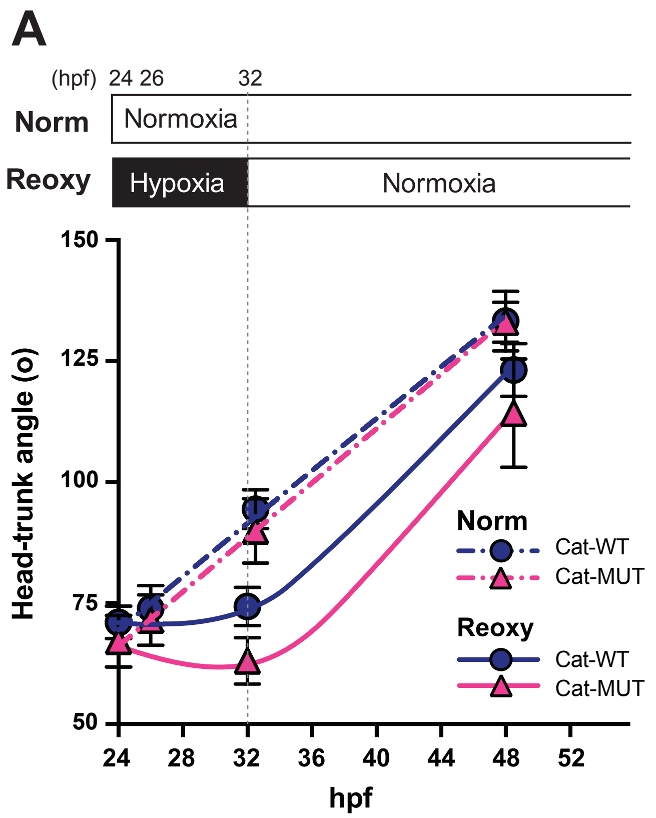
A
(hpf) 24 26 32
Norm
Normoxia
Reoxy
Hypoxia
Normoxia
Head-trunk angle (o)
150
125
100
75
50
24 28 32 36 40 44 48 52
hpf
Norm
Cat-WT
Cat-MUT
Reoxy
Cat-WT
Cat-MUT

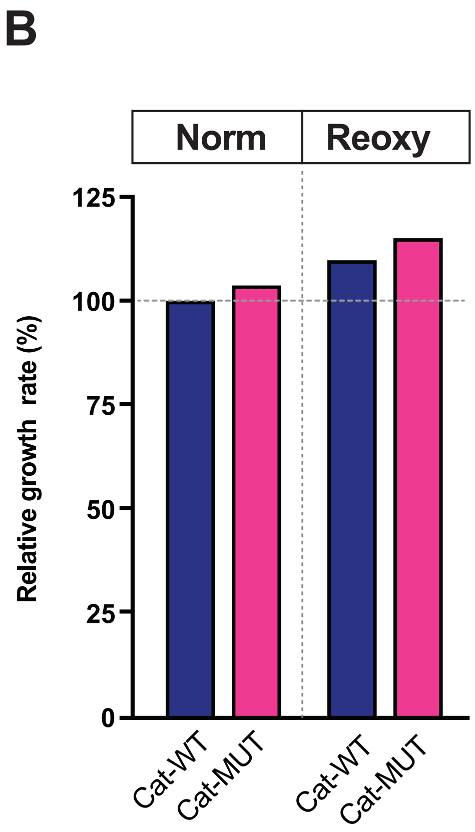
B
Norm
Reoxy
Relative growth rate (%)
125
100
75
50
25
0
Cat-WT
Cat-MUT
Cat-WT
Cat-MUT

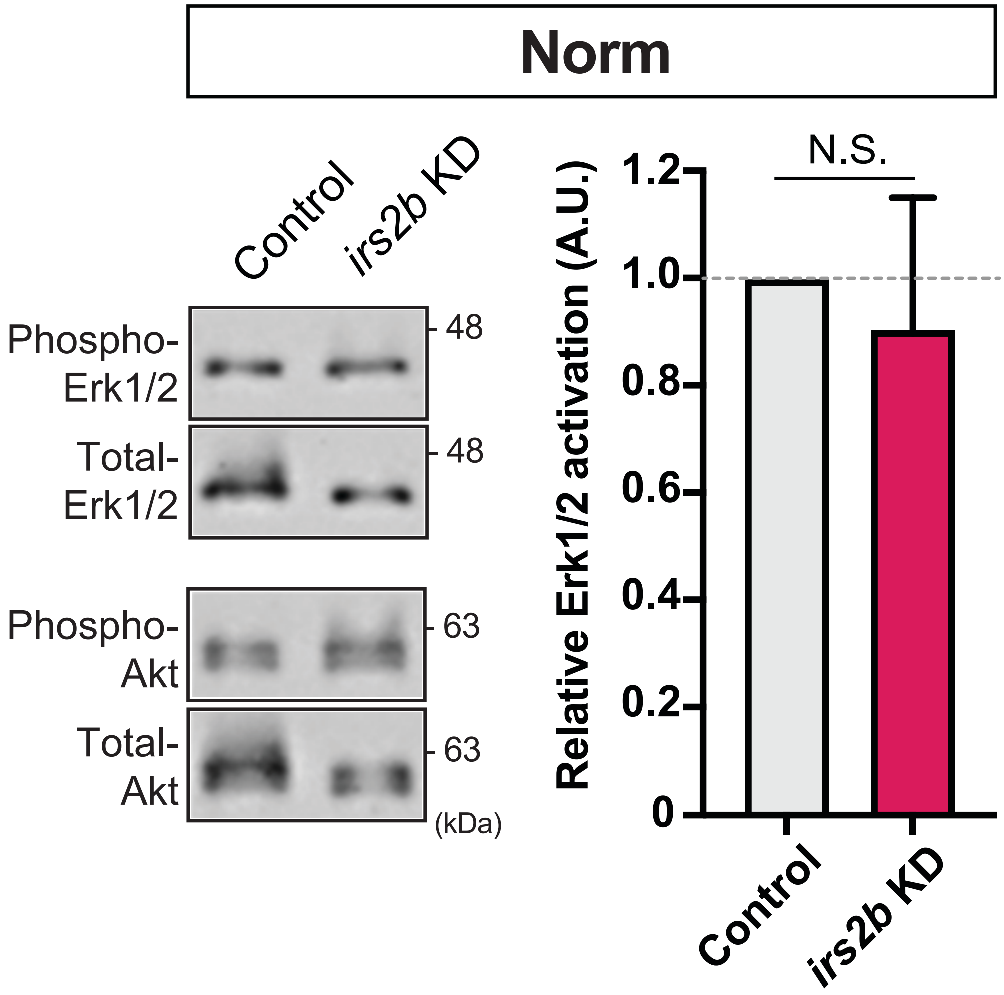
Norm
Control
irs2b KD
Phospho-Erk1/2
48
Total-Erk1/2
48
Phospho-Akt
63
Total-Akt
63
(kDa)
N.S.
Relative Erk1/2 activation (A.U.)
1.2
1.0
0.8
0.6
0.4
0.2
0
Control
irs2b KD
